# Display of the HIV envelope protein at the yeast cell surface for immunogen development

**DOI:** 10.1371/journal.pone.0205756

**Published:** 2018-10-18

**Authors:** Elizabeth Mathew, Hong Zhu, Sara M. Connelly, Mark A. Sullivan, Matthew G. Brewer, Michael S. Piepenbrink, James J. Kobie, Stephen Dewhurst, Mark E. Dumont

**Affiliations:** 1 Department of Biochemistry and Biophysics, University of Rochester Medical Center, Rochester, NY, United States of America; 2 Department of Microbiology and Immunology, University of Rochester Medical Center, Rochester, NY, United States of America; 3 Infectious Diseases Division, University of Rochester Medical Center, Rochester, NY, United States of America; University of Nebraska-Lincoln, UNITED STATES

## Abstract

As a step toward the development of variant forms of Env with enhanced immunogenic properties, we have expressed the glycoprotein in the yeast surface display system in a form that can be subjected to random mutagenesis followed by screening for forms with enhanced binding to germline antibodies. To optimize the expression and immunogenicity of the yeast-displayed Env protein, we tested different approaches for cell wall anchoring, expression of gp120 and gp140 Env from different viral strains, the effects of introducing mutations designed to stabilize Env, and the effects of procedures for altering N-linked glycosylation of Env. We find that diverse forms of HIV envelope glycoprotein can be efficiently expressed at the yeast cell surface and that gp140 forms of Env are effectively cleaved by Kex2p, the yeast furin protease homolog. Multiple yeast-displayed gp120 and gp140 proteins are capable of binding to antibodies directed against the V3-variable loop, CD4 binding site, and gp41 membrane-proximal regions, including some antibodies whose binding is known to depend on Env conformation and N-linked glycan. Based on antibody recognition and sensitivity to glycosidases, yeast glycosylation patterns partially mimic high mannose-type N-glycosylation in mammalian cells. However, yeast-displayed Env is not recognized by some anti-Env antibodies sensitive to quaternary structure, suggesting either that the displayed protein exists in a monomeric state or that for these antibodies, yeast glycosylation in certain regions hinders recognition or access. Consistent with studies in other systems, reconstructed predicted unmutated precursors to anti-Env antibodies exhibit little affinity for the yeast-displayed envelope protein.

## Introduction

Despite the success of anti-retroviral drugs in treating HIV-infected individuals and slowing the spread of infection, these drugs remain unaffordable by much of the world and are subject to side effects and development of resistance. The use of a vaccine against HIV is generally viewed as the most effective long term approach for controlling or eradicating the worldwide AIDS pandemic [1]. To date, however, intensive efforts to develop an effective AIDS vaccine (more than 200 clinical trials [2]) have failed to yield an effective immunogen, aside from a single trial showing only a modest protective effect [3].

The potential feasibility of effective vaccination is suggested by the observation that some infected individuals develop antibodies that can neutralize a broad array of virus strains *in vitro* and can prevent virus transmission in animal models. In addition, an attenuated form of the related simian SIV virus provides protection against infection [4] and passive immunization using broadly neutralizing antibodies (bnAbs) derived from infected humans is now an active area of research [5]. All known broadly neutralizing antibody responses against HIV are targeted to the viral envelope glycoprotein (Env) that is initially translated as a 160 kDa product (gp160), then cleaved by cellular furin proteases into two chains, the gp120 and gp41 glycoproteins. The difficulty of developing a protective vaccine appears to arise, in part, from the high rate of mutagenesis of the HIV virus, the masking function of the viral glycan shield, the sequestered nature of conserved structures involved in cell binding and entry, and the very low affinity of germline antibodies for neutralizing epitopes on Env. In view of these factors, there has been intensive investigation of the sequences and structural features mediating interactions between Env and known neutralizing antibodies, resulting in identification of several targets of bnAbs. These include the CD4 receptor binding site [6, 7], certain glycans on the Env surface [8], sites on gp41 that are likely to be involved in membrane fusion with target cells [9], and sites on the V2 and V3 loops [10] and V1 and V2 loops [11] of gp120 that appear to depend on a particular quaternary structure of the envelope protein.

In cases where it has been possible to identify germline precursors of broadly neutralizing antibodies, the precursors display little [12] or no [13–15] affinity for Env. This has led to an interest in designing immunogens that more efficiently engage germline B cell receptors. However, the current level of structural characterization of Env-antibody complexes and the existing capabilities of procedures for “rational” protein engineering, may not be sufficient to allow design of an immunogen that can elicit the desired protective responses. An alternative (or complement) to the rational design of more effective immunogens is the use of random mutagenic or gene shuffling approaches followed by screening for variant forms of Env with a desired set of antigenic/immunogenic properties. This approach has previously been applied to in several systems: 1) Random libraries of short peptides comprising potential epitopes from Env have been expressed in bacterial cells and displayed on phage particles [16, 17]; 2) Mutated versions of a scaffolded form of gp120 lacking variable loop regions have been displayed on the surface of yeast cells in order to identify variant forms with enhanced binding to precursor forms of anti-CD4 binding site antibodies [18–21]; 3) Mutated forms of gp140 have been displayed on yeast cells in a screen for optimized expression, stability, and binding of mature anti-CD4 binding site antibodies [22]; and 4) Variant forms of gp120 have been expressed in cultured mammalian cells [23] and used to screen for variants with enhanced binding to precursor forms of anti-variable loop bnAbs [24].

Previous screening in the yeast display system for variant forms of Env with enhanced affinity for bnAb precursors used scaffolded versions of HIV Env lacking variable loops. While these efforts have, in fact, yielded altered versions of Env displaying enhanced affinities for germline precursor forms of bnAbs [19, 20], we wished to extend the approach to intact forms of Env that would more completely mimic the full range of possible interactions between the glycoprotein and antibody precursors. As an initial step toward this goal, we describe here the optimization of the display of antigenically active HIV envelope protein at the yeast cell surface and characterization of the binding of different forms of the displayed protein to precursor and mature forms of anti-HIV antibodies. These studies provide a basis for the ongoing utilization of the yeast-display system for screening randomly mutagenized libraries of Env proteins to identify variant forms of Env with enhanced abilities to activate B cells expressing precursor forms of bnAbs.

## Results and discussion

**Fig 1:**
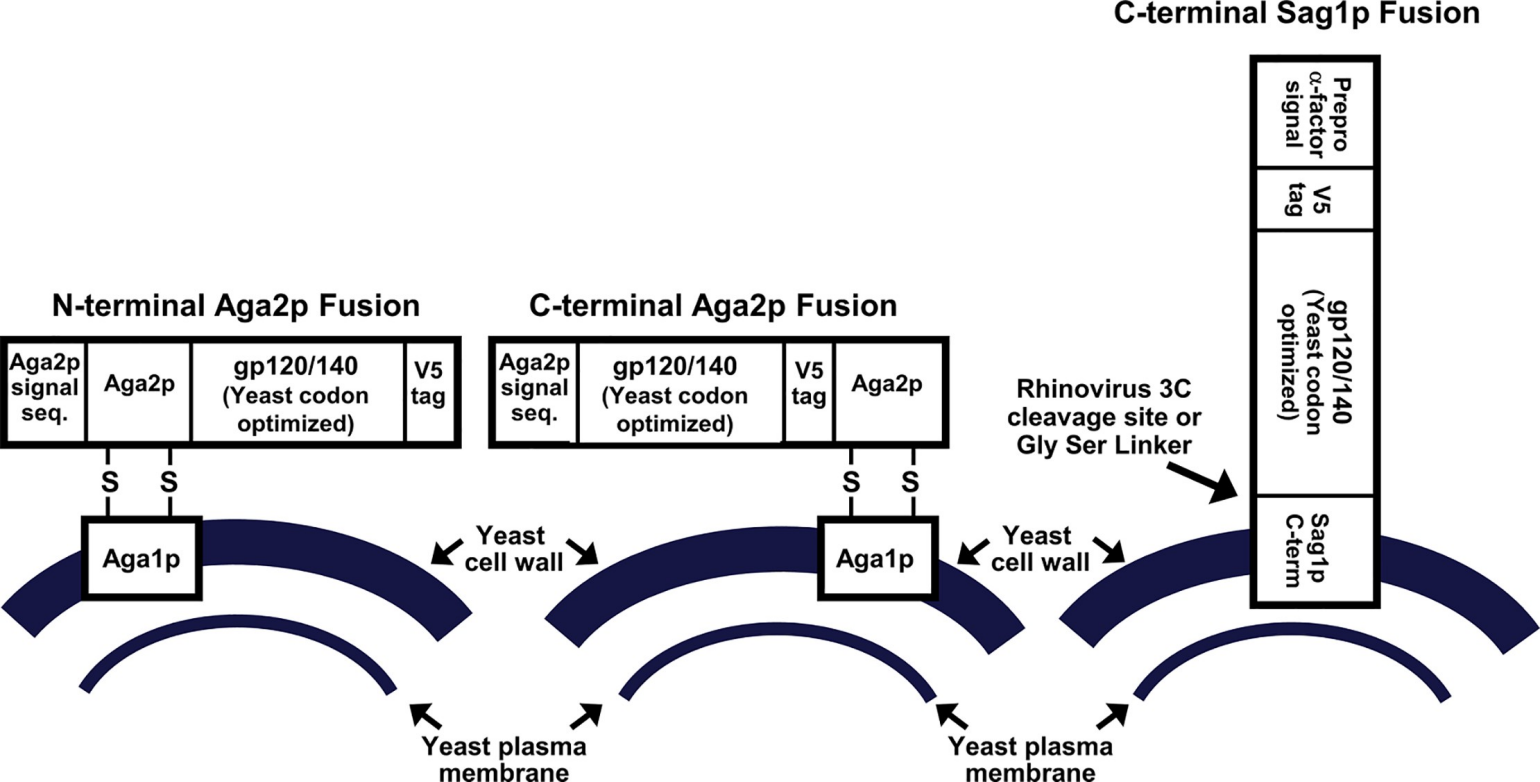
Schematic diagram of different systems of yeast display of HIV envelope glycoprotein.

To optimize expression of the antigenically active Env on the yeast cell surface, we varied the geometry of the fusion constructs (see Fig 1), used Env proteins from different viral strains, and varied plasmid constructs and induction conditions affecting expression levels, the extent of glycosylation, and the oligomeric state of the expressed envelope. The goal was to achieve maximal binding of diverse anti-Env antibodies as detected using fluorescent anti-human secondary antibodies, while also achieving the highest proportion of expressing vs. non expressing cells, since, even in selective medium, yeast commonly undergo loss of plasmids, especially if they encode toxic proteins [25]. Thus, we synthesized yeast codon-optimized genes encoding different length forms of Env derived from four different HIV strains and containing various combinations of previously-identified mutations thought to stabilize gp41-gp120 interactions and trimeric forms of the protein (see S1 Fig). Based on the galactose-inducible expression system commonly used for yeast display, we established a protocol for Env expression using initial growth in unbuffered synthetic medium with raffinose as a carbon source (to relieve glucose repression of the *GAL* promoter), followed by growth in raffinose medium buffered at pH 6 (to optimize Env folding and expression), followed, in turn, by growth in galactose-containing medium, also buffered at pH 6. The final growth and induction was conducted at 20°C, which we found, in some cases, enhances the ability of the expressed Env protein to bind antibodies.

### Fusion strategies

**Fig 2:**
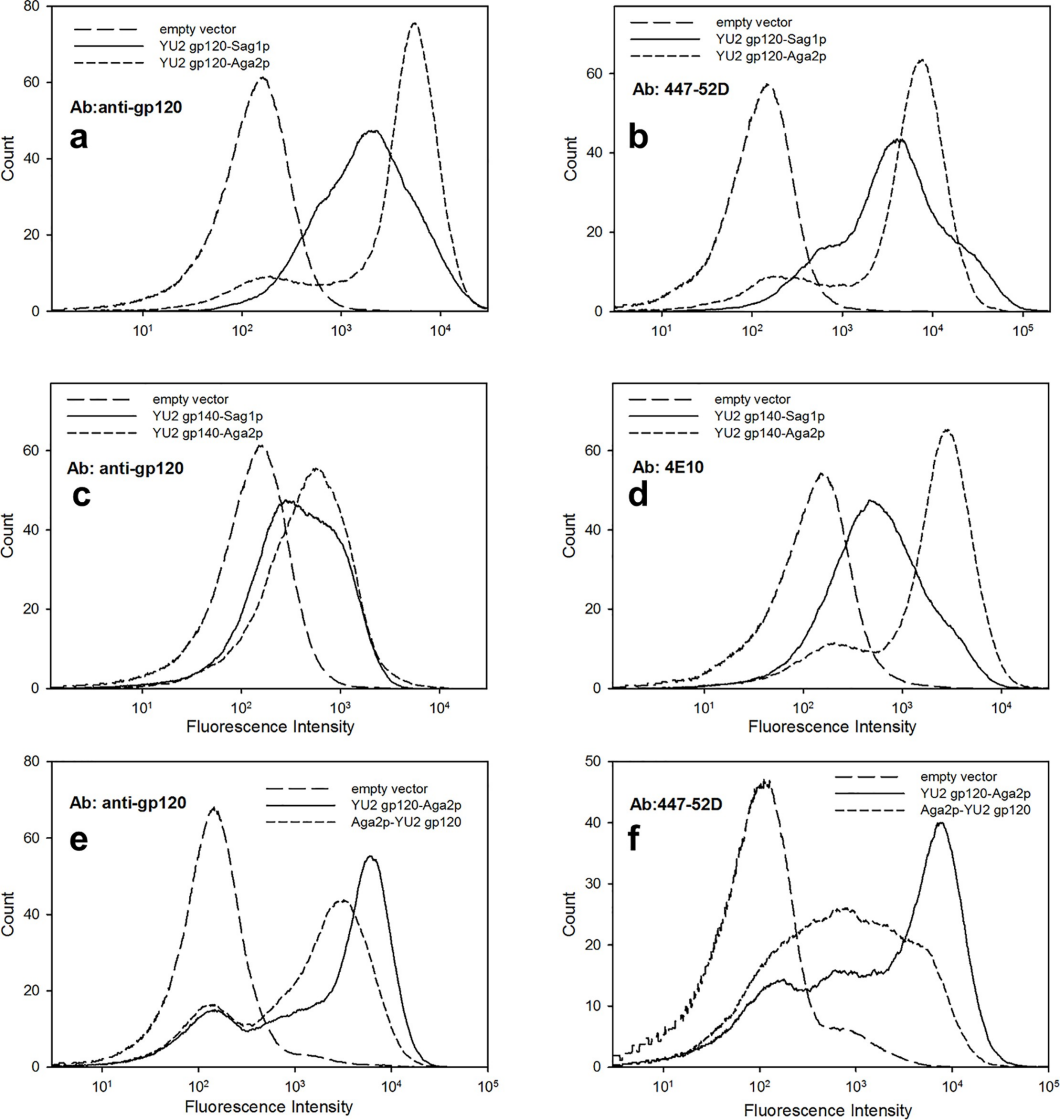
Comparison of different systems for yeast surface display of HIV Env. (a),(b) Flow cytometry histogram showing the fluorescence analysis of ~10,000 cells expressing gp120 from viral strain YU2 fused at its C-terminal (with a mutated furin cleavage site) to Sag1p or Aga2p upon binding to anti-gp120 polyclonal Ab (a) (66 nM) and anti-V3 loop antibody 447-52D (b) (30 nM). (c),(d) Fluorescence analysis of ~10,000 cells expressing gp140 from viral strain YU2 fused at its C-terminal to Sag1p or Aga2p upon binding to anti-gp120 polyclonal Ab (c) (66 nM) and anti-MPER antibody 4E10 (d) (16 nM). (e),(f) Fluorescence analysis of ~10,000 cells expressing gp140 from viral strain YU2 fused at either its N-terminal or C-terminal to Aga2p upon binding to anti-gp120 polyclonal Ab (e) (66 nM) and anti-V3 loop antibody 447-52D (e) (90 nM). For panels (a)–(d), incubation with the secondary antibody was performed at 4°C and cells were kept on ice prior to flow cytometry. For panels (e) and (f), cells were maintained at room temperature during the incubation with secondary antibody and prior to flow cytometry.

We investigated strategies for anchoring Env to the yeast cell wall in which Env is either attached directly to the yeast cell wall α-agglutinin Sag1p as a C-terminal fusion [26–28], or fused to the secreted protein Aga2p either as an N- or C- terminal fusion (See Fig 1). Fusion to Aga2p forms the basis for the most commonly-used version of yeast surface display in which the **a**-agglutinin Aga2p is linked via disulfide bridges to the outer surface of the cell wall-linked agglutinin subunit Aga1p, which is overexpressed in the yeast host strain [29, 30]. For Sag1p constructs, the endogenous signal sequence of Env was replaced by a sequence derived from the yeast α-mating factor that is commonly used for heterologous secretion in yeast. All Aga2p fusions used the signal sequence from Aga2p, either in its normal position in Aga2p (in constructs where Aga2p is fused to the N-terminal of Env), or as a replacement for the endogenous Env signal (in constructs where Aga2p is fused to the C-terminal of Env.) Expression of gp120 from HIV strain YU2 was greater for the C-terminal Aga2p fusion, compared with C-terminal Sag1p fusion, as assayed by binding to polyclonal anti-gp120 antibody (Fig 2A). In addition, binding of several human anti-Env antibodies to the gp140 form of YU2 Env was greater for the Aga2p-fusion than for the Sag1p-fusion (Fig 2B–2D). Thus we have focused subsequently on Aga2p fusion constructs.

We also compared N- and C-terminal fusion strategies for Aga2p fused to gp120. Fusion of Aga2p at the C-terminal provides higher expression levels based on binding of polyclonal anti-gp120 antibody and antibody 447-52D (Fig 2E and 2F). Since Env is normally anchored to cell membranes via the transmembrane segment near the C-terminal of the gp41 moiety, C-terminal fusion to Aga2p mimics its orientation at the viral membrane. For the C-terminal Env-Aga2p fusions, the native Env signal sequence was replaced with a modified version of the signal sequence from Aga2p that has an Ala-Gly peptide spacer sequence after the signal peptide cleavage site to promote efficient cleavage of the signal peptide [31].

### Effects of stabilizing mutations on antibody binding to Env constructs

In accordance with the goal of maintaining the full repertoire of potential interactions with antibodies and B-cell receptors that occurs during bnAb development, we expressed full-length gp120 and gp140 regions. All tested gp120-Aga2p fusion constructs contained substitutions of serine for all arginine residues in the sequence RRVVQREKR, corresponding to residues 503–511 (HXB2 numbering) of gp120 in order to maintain anchorage of gp120 to the yeast cell wall by preventing cleavage between the gp120 and Aga2p by the yeast furin analog Kex2p. As noted, below, this was not completely effective in blocking Kex2p cleavage.

We also tested the effects of mutations in Env previously shown to promote the formation of stable trimers [32] and interactions of yeast-displayed gp140 constructs with neutralizing antibodies [22]. In particular, we compared the following types of Env constructs: 1) “unmodified” forms based directly on Env sequences of natural viral strains; 2) “SOSIP” constructs containing substitutions previously engineered to stabilize gp140 complexes in envelopes from strains JRFL [32] and BG505 [33]. These include the substitution I559P, introduction of the N-glycosylation site at N332 (in Env from viral strain BG505 only, as the other strains we used already contain Asn at position 332), as well as the disulfide-forming substitutions A501C and T605C. 3) “dSOSIP” constructs containing the SOSIP mutations plus additional substitutions shown by Grimm *et al*. to enhance expression and antibody binding of the SOSIP version of the JRFL envelope at the yeast surface. These include replacement of the furin cleavage site between gp120 and gp41 with a sequence optimized for cleavage by Kex2p, removal of a possible alternative Kex2p cleavage site, and replacement of the hydrophobic residues in the Env fusion peptide by a more hydrophilic sequence based on sequences from other viral strains [22]. 4) “dsm” constructs containing substitutions identified by Grimm *et al*. through random mutagenesis and screening [22]) that promote expression and antibody binding in the yeast display system. Mutations corresponding to SOSIP and dsm constructs in envelope proteins from different viral strains were identified based on the sequence alignment shown in S1 Fig.

We created C-terminal Aga2p fusion constructs based on envelope proteins from four different HIV-1 strains (sequences listed in S1 Fig). The YU2, BG505, and JRFL strains were used because they serve as the basis for a number of widely-used constructs. We also examined constructs based on Env of the QH0692 strain, which has been reported to be capable of weakly binding to a precursor form of the anti-V3 loop antibody 447-52D [34].

**Fig 3:**
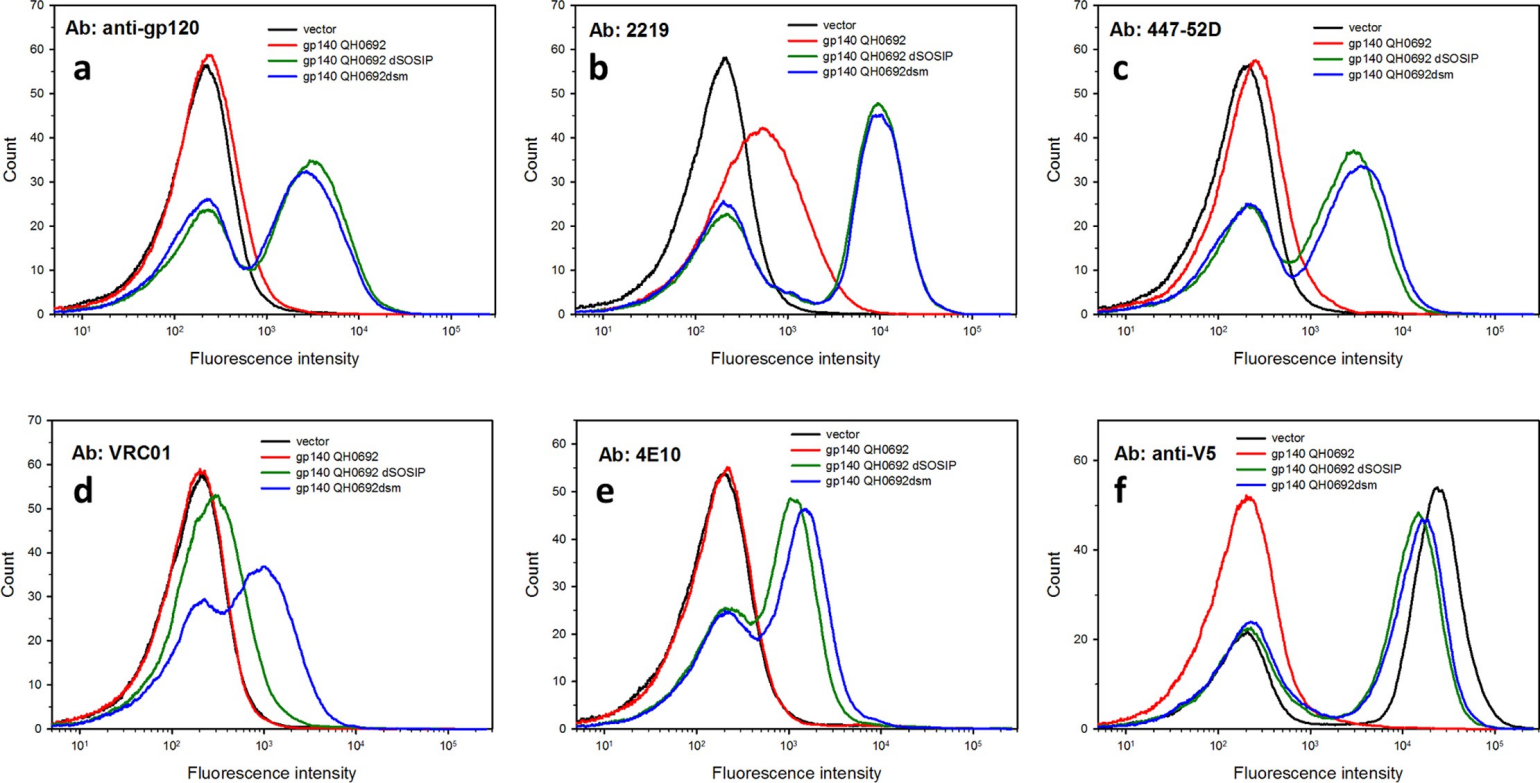
Modified versions of yeast surface displayed gp140 from viral strain QH0692 bind anti-HIV antibodies more efficiently than unmodified forms. The figure shows a fluorescence analysis of ~10,000 cells following incubation with the indicated primary antibodies and fluorescent secondary antibody. (a) anti-gp120 polyclonal antibody (~66 nM); (b) anti-V3 loop antibody 2219 (320 nM) (c) anti-V3 loop antibody 447-52D (90 nM) (d) anti-CD4 binding site antibody VRC01 (340 nM); (e) anti-MPER region antibody 4E10 (200 nM); (f) anti-V5 epitope antibody (13 nM). (The empty vector expresses a V5-Aga2p fusion). Secondary antibody incubation and flow cytometry were performed at room temperature.

The stabilizing mutations comprising dsm versions of Env gp140 provided clear enhancements of antibody binding to all four tested gp140 constructs derived from different viral strains (Fig 3 and S2 Fig). However, the extents of these enhancements depended on the particular viral strain from which the Env was derived and on the particular antibody being tested. For the gp140 derived from the QH0692 strain, the dSOSIP version bound better than unmodified QH0692 Env to all tested antibodies and, for all tested antibodies except VRC01, there was little additional enhancement of antibody binding upon introduction of the dsm substitutions (Fig 3A–3F). VRC01 binds substantially better to the dsm form of QH0692 gp140 than to the dSOSIP form (Fig 3D), consistent with the fact that the dsm mutations were originally identified based on their ability to enhance binding to VRC01 [22]. Incorporation of dsm substitutions into gp140s derived from viral strains JRFL, BG505, and YU2 also enhanced antibody binding and expression (based on anti-gp120 binding) compared to binding to unmodified forms of gp140 (Figures a-k in S2 Fig) or, for BG505, compared to the original SOSIP version lacking changes at the cleavage site and fusion peptide (Figures l-o in S2 Fig).

### Antibody binding specificities

**Fig 4:**
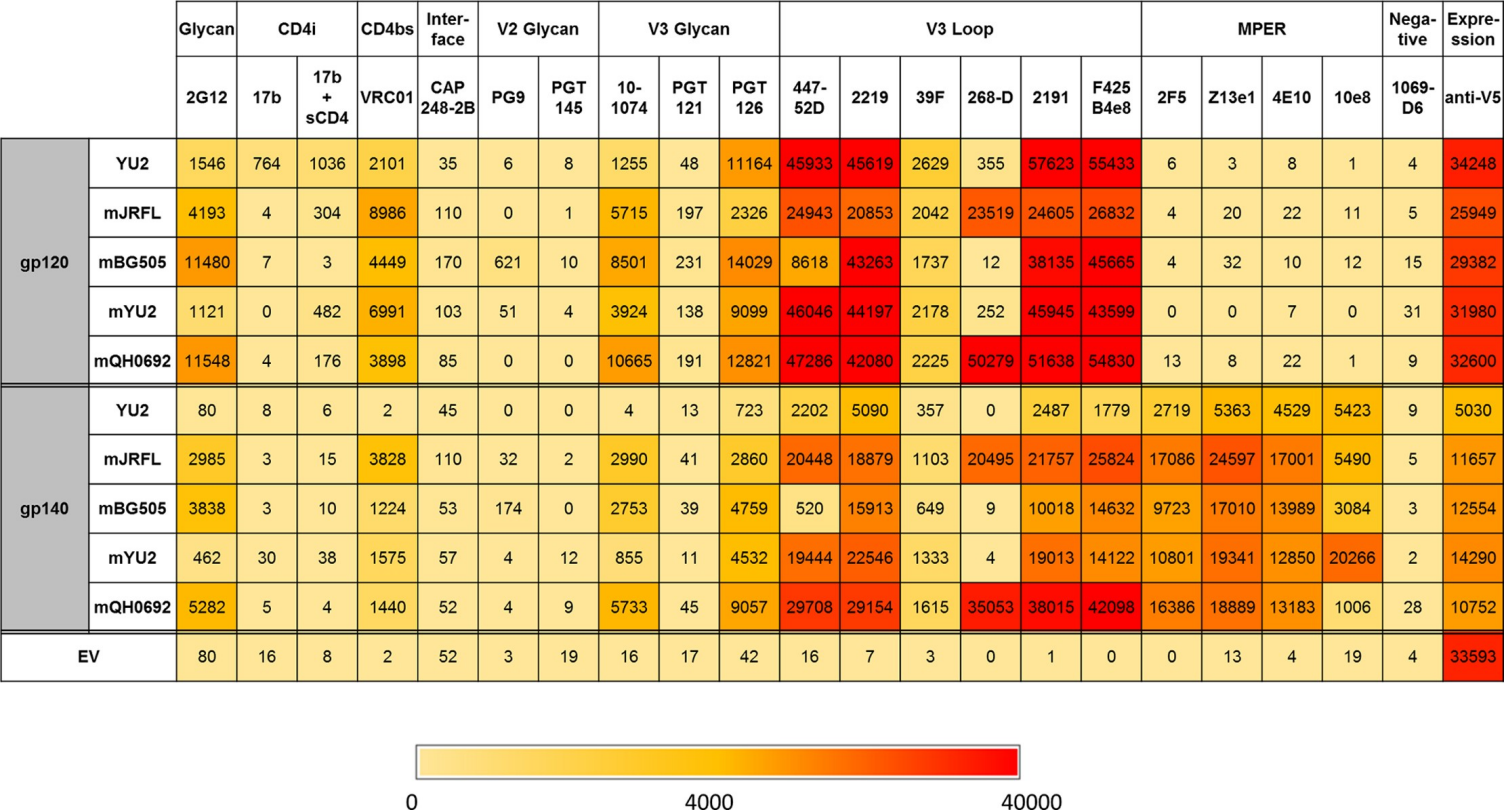
Comparison of binding of various anti-HIV bnAbs to yeast surface displayed HIV envelope from 4 viral strains. The “m” preceding the HIV strain name indicates the modified dsm version. The concentrations of the various antibodies are as follows: anti-V5, 20 nM; anti-gp120 polyclonal, 60 nM; all others, 100 nM. Where indicated, soluble CD4 (sCD4) was used at a concentration of 100 nM. If the mean fluorescence after subtraction of the mean fluorescence of secondary plus cells was negative, it was assigned a value of zero. The color scale is non-linear. Secondary antibody incubations were performed at 4°C and cells were maintained on ice prior to flow cytometry. Further details of these binding determinations are tabulated in S1 and S2 Tables.

A compendium of the binding of mature forms of anti-HIV antibodies to different yeast-displayed forms of Env is presented in Fig 4. The figure lists the mean fluorescence intensities of cells treated with a single concentration of each antibody (see legend to Fig 4) followed by a secondary Alexa_647_-conjugated anti-human antibodies, detected using flow cytometry. Overall expression levels of different constructs containing Aga2p as a C-terminal fusion were also assayed by flow cytometry based on the binding of mouse antibodies recognizing the V5 epitope present at the junction of Env and Aga2p, followed by Alexa_633_-conjugated anti-mouse secondary. The highest levels of fluorescence detected were for anti-V5 binding to an “empty vector” Aga2p construct (yeast strain A4793) that contains the V5 epitope but no envelope protein. The fact that fluorescence levels for all other samples were lower than this shows that they had not surpassed the dynamic range of the detection system. (A full tabulation of the binding results is provided in S1 Table)

In evaluating antibody binding to Env constructs, we considered the statistical significance of the observed mean fluorescence intensity, compared to binding of the same antibody to cells expressing empty vector. (S2 Table provides a tabulation of the p values for each antibody/Env construct combination). 16 of the 19 anti-Env antibodies tested in Fig 4 exhibited binding to at least one yeast-displayed Env construct characterized by a p value of less than 0.01 and no significant binding was observed to the human negative control antibody 1069-D6.

Substantial variations were observed in comparing the binding of the tested antibodies to dsm forms of gp120 and gp140 from different viral strains. Some of these variations likely stem from different levels of glycoprotein expression. For example, based on binding of anti-V5 epitope antibodies (Fig 4), levels of expression of gp120 constructs are higher than those of the corresponding forms of gp140. The unmodified gp120 form of YU2 binds anti-V5 antibodies at a levels approximately 7-fold higher than does the unmodified YU2 gp140 (p < 0.001). The dsm forms of gp120 from different viral strains exhibit levels of anti-V5 antibody binding that are 2–3 times higher than those of the corresponding gp140 forms (p < 0.001 in pairwise comparisons). Consistent with these observations, levels of binding of most antibodies (except for gp41-directed antibodies) were uniformly higher for gp120 constructs than for the corresponding gp140 constructs. However, the variations seen in binding of human monoclonal anti-HIV antibodies to different yeast-displayed Env constructs are much greater than the variations in anti-V5 binding, indicating that most of the variation in anti-HIV antibody binding is due to diversity in the sequences or conformations of the epitopes for these antibodies.

Fig 4 (together with Fig 3 and S2 Fig) shows that the dsm form of YU2 gp140 (but not gp120) always bound higher levels of antibodies than the unmodified form. For example, binding of the four tested anti-MPER antibodies to the dsm form of YU2 gp140 resulted in fluorescence levels that averaged 3.6 (± 0.2) times those of the unmodified YU2 gp140. For anti-V3 loop antibodies, the fluorescence values for the dsm form of YU2 gp140 averaged 6.6 (± 1.0) times the values for the unmodified YU2 gp140. Thus, the dsm mutations enhance the binding of several different classes of antibodies to different extents, despite the fact that these mutations were isolated based on their ability to promote binding of only the anti-CD4 binding site antibody VRC01 [22]. Much of this effect appears to be due to increased expression of the dsm forms, since anti-V5 binding to the YU2 gp140dsm resulted in a fluorescence value three times higher than that for the unmodified YU2 gp140 (p < 0.001).

#### Binding of anti-CD4 binding site antibodies to yeast-displayed Env

As shown in Fig 4, we detected binding of the anti-CD4 binding site antibody VRC01 [7] to all of the tested yeast-displayed constructs except the unmodified YU2 gp140. While, in contrast to some previous reports [21, 22], VRC01 bound to surface-displayed unmodified intact YU2 gp120, incorporation of the dsm mutations [22] enhanced VRC01 binding to both the gp120 and gp140 versions of the YU2 envelope.

**Fig 5:**
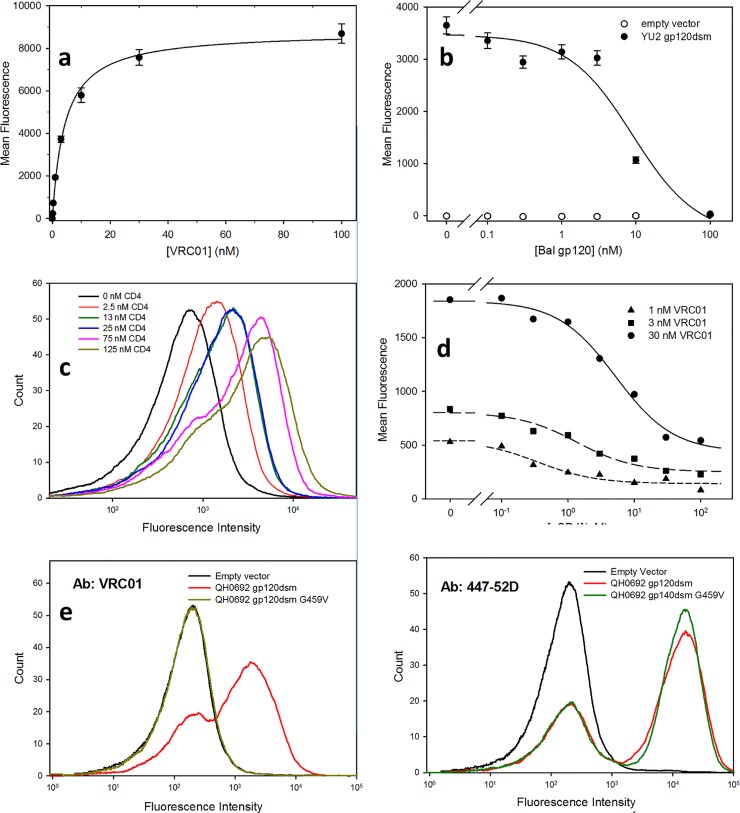
Characterization of the CD4 binding site of yeast-displayed gp120. (a) Saturation binding of anti-CD4 binding site antibody VRC01 to YU2 gp120dsm (apparent K_d_; 4.3 ± 0.2 nM). (b) Competition of VRC01 (3 nM) binding to YU2 gp120 dsm by increasing concentrations of gp120 from the Bal HIV strain. Competition was characterized by a log(IC_50_) of 0.96 ± 0.04 (nM) corresponding to a K_i_ of 5 nM for VRC01 binding to the purified gp120. (c) Binding of a soluble form of CD4 to YU2 gp120 (d) Competition of binding of VRC01 at the indicated concentrations of soluble CD4 to gp120 (logIC50s: -0.45 ± 0.20 (1 nM VRC01); 0.13 ± 0.16 (3 nM VRC01); 0.72 ± 0.09 (30 nM VRC01); (error estimates from least squares fit)), K_i_ for VRC01 binding to sCD4, 0.58 ± 0.15 nM (error estimated from measurements at three antibody concentrations)) (e) The G459V substitution in the CD4 binding site abolishes VRC01 (70 nM) binding to QH0692 gp120dsm. (f) The G459V substitution does not affect binding of gp120dsm to the anti-V3 loop antibody 447-52D (30 nM). (For panels (a), (b), (d), secondary antibody incubations performed at 4°C and cells kept on ice until flow cytometry. For panels (c), (e), (f), cells were maintained at room temperature.).

Several results indicate that VRC01 recognizes a native-like CD4bs epitope on yeast-displayed Env: 1) In saturation binding experiments with yeast cells expressing YU2 gp120dsm, VRC01 binds with an apparent K_d_ of 4.3 ± 0.2 nM (Fig 5A). 2) Binding of VRC01 to yeast cells expressing YU2 gp120dsm can be competed by the addition of gp120 (Bal strain) purified from HEK293 cells (Fig 5B). 3) Binding of a purified soluble form of CD4 [35, 36] to yeast-displayed YU2 gp120 could be detected using anti-CD4 antibodies together with secondary fluorescent anti-human antibodies (Fig 5C). 4) Purified soluble CD4 competes with the binding of VRC01 to yeast expressed YU2 gp120 (Fig 5D). 5) A mutant form of QH0692 gp120dsm containing the substitution G459V at a position involved in a critical intramolecular hydrogen bond with VRC01 [21, 37, 38] exhibits a nearly complete loss of VRC01 binding (Fig 5E), while retaining normal binding of the anti-V3 loop antibody 447-52D (Fig 5F).

The antibody 17b recognizes an epitope that partially overlaps the co-receptor binding site on Env that is exposed by a conformational change when Env binds CD4 [39, 40]. Weak binding of 17b to unmodified YU2 gp120 (but no other displayed form of gp120 or gp140) was detected in the absence of CD4. Addition of purified soluble CD4 resulted in increased binding to the dsm forms of JRFL, YU2, and QH0692 gp120, indicating that at least some subset of the displayed gp120dsm forms is capable of undergoing a CD4-dependent conformational change. However, the presence of the soluble CD4 provided little increase in binding of 17b to the unmodified yeast-displayed YU2 gp120 (see Fig 4 and S1 and S2 Tables).

#### Binding of antibodies direct against variable loops of yeast-displayed Env

**Fig 6:**
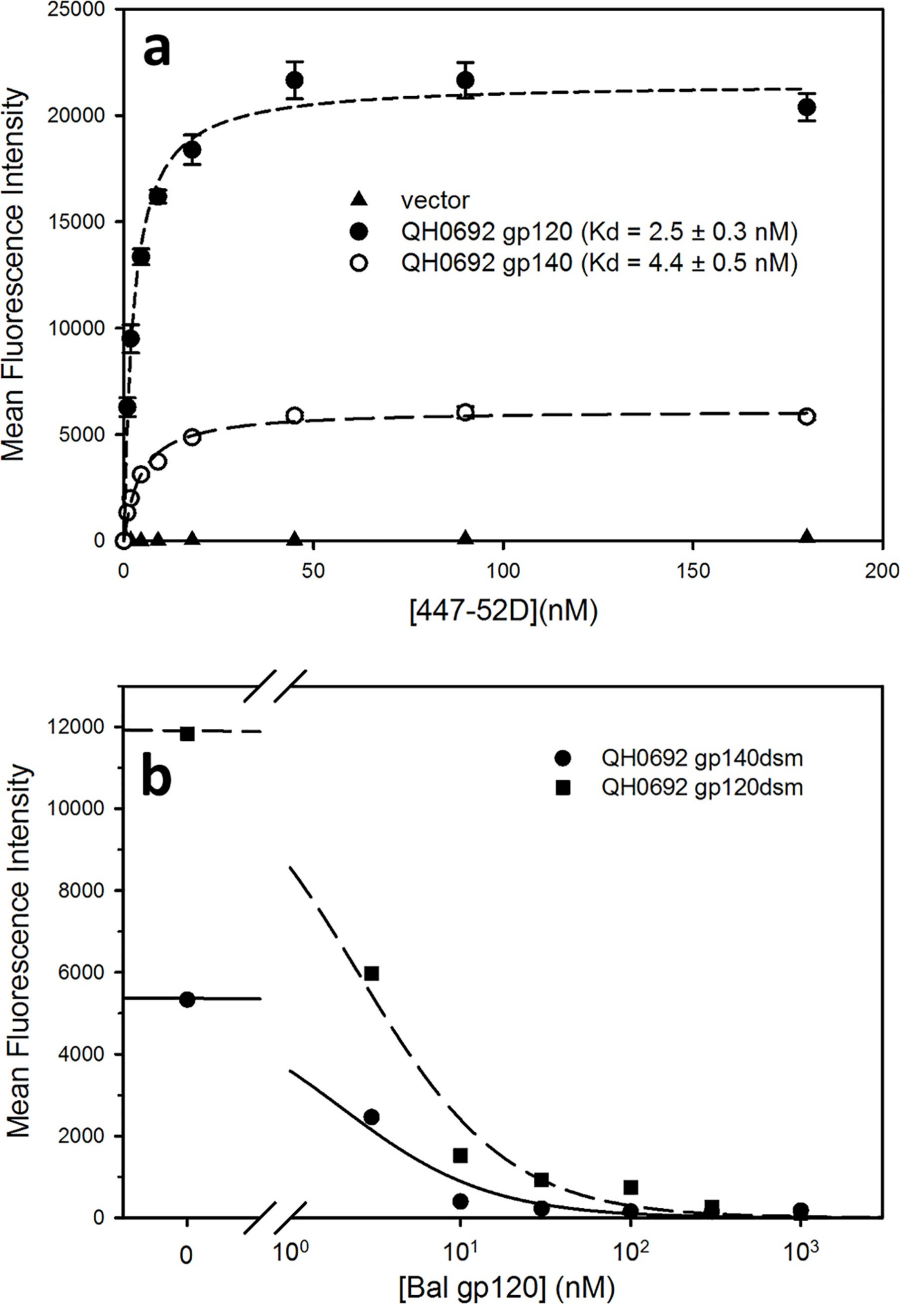
Binding of anti-V3 antibody 447-52D to Env from viral strain QH0692. a) Saturation binding of anti-V3 antibody 447-52D to QH0692 gp140dsm and gp120dsm. b) Binding of 447-52D (3 nM concentration) is competed by purified gp120 from viral strain Bal. The logIC_50_s for competition are 0.30 ± 0.09 and 0.40 ± 0.07 for gp140 and gp120, respectively, corresponding to K_i_s of 1.2 and 1.1 nM for binding of 447-52D to the purified Bal gp120.

In contrast to previous studies of yeast-displayed scaffold forms of gp120 [19, 21, 41], anti-loop region-antibodies bound effectively to the yeast-displayed full-length gp120 and gp140 constructs. Four of these antibodies, 447-52D, 2191, 2219, and F425B4e8, exhibited the strongest binding to yeast-displayed Env among all the tested anti-HIV antibodies. Dissociation constants for 447-52D binding to the QH0692 gp120dsm and gp140dsm constructs were determined by saturation binding experiments to be 2.5 ± 0.3 and 4.4 ± 0.5 nM, respectively, with approximately 4-fold greater number of sites per cell for the gp120 form (Fig 6A). These values are much lower than those reported for binding of 447-52D and related anti-V3 antibodies to V3 peptides and purified recombinant gp120 determined by ELISA [42], by isothermal titration calorimetry [43], or by saturation binding to a mammalian cell surface display system [44], but are higher than the K_d_ reported for 447-52D binding to V3-related synthetic peptides determined by surface plasmon resonance [45] and are much higher than the apparent K_d_ for purified QH0692 gp140 trimers determined by ELISA. The binding of 447-52D to the gp120dsm and gp140dsm forms of QH0692 Env was effectively competed by purified Bal strain envelope produced in HEK293 cells (Fig 6B).

Antibody 447-52D recognizes a linear epitope in the V3 loop, as well as possible additional conformational epitopes [46–48]. Consistent with reports that it most effectively binds and neutralizes viruses in which the sequence G^312^PGR^315^ is present at the tip of the V3 loop [49–52], we find that binding of 447-52D to the gp120dsm and gp140dsm forms of BG505 Env, which contain the sequence G^312^PGQ^315^ in the V3 loop, is considerably weaker than binding to stabilized envelope constructs from strains YU2, JRFL, and QH0692 that contain the sequence G^312^PGR^315^. In contrast to the findings for 447-52D, we find that antibodies 2191, 2219, and F425B4e8, which also recognize linear epitopes in V3, show little or no preference for binding to yeast-displayed forms of Env containing the GPGR sequence, consistent with reports that these antibodies can interact with forms of Env containing GPGQ sequences [48, 53–55].

Anti-V3 loop antibody 268-D exhibits a strong preference for binding to gp120 and gp140 forms of Env from viral strains JRFL and QH0692 with little or no binding to forms of Env derived from YU2 or BG505. 268-D recognizes a peptide corresponding to the sequence H^310^IGPGR^315^ [42, 49], and based on a crystal structure with a peptide encompassing this region, interacts most strongly with three basic residues in the V3 crown K305, H308, and R315 [56]. Thus, the failure to bind well to BG505 can be explained both by the substitution of arginine for H308 and the presence of GPGQ^315^. Failure to bind YU2 forms of Env can be explained by the substitution of asparagine for H308 in this strain.

Antibody 39F, which has been reported to primarily recognize the sequence K^305^SI^307^, binds moderately to all tested forms of gp120 and gp140. The failure to discriminate between envelopes of different strains is consistent with the presence of the KSI sequence in all the tested Env constructs.

Yeast-displayed gp120s and gp140s (particularly the dsm forms) also bound antibodies reported to be directed against combinations of loop peptide and glycans, including 10–1074 [57–60] and PGT126 [61], directed against the V3 loop region. This suggests that patterns of glycosylation in yeast may, be similar enough to certain patterns in mammals (such as high mannose N-glycosylation) to allow antibody recognition. All of the tested constructs in Fig 4 contain the potential N-glycosylation site at position 332 that has been implicated as a recognition site for this class of V3-loop/glycan-directed bnAbs [60, 62]. Both antibodies 10–1074 and PGT126 (which are from the same lineage) are reported to preferentially bind high mannose glycosylation at position 332 (which, compared to complex glycosylation, is likely to be more similar to yeast glycosylation patterns) [60, 61]. PGT126 has also been previously reported to bind to endogenous yeast glycoproteins from strains with modified glycosylation pathways [63], though we detected no significant background binding to normal strains that do not express Env constructs (Fig 4).

Antibody 10–1074 binds more weakly to modified and unmodified forms of YU2 than it does to envelopes from other viral strains. In view of the high degree of conservation of sequence in the V3 region in comparing the tested Env constructs, low binding to YU2 derivatives could be the result of the presence of an uncharged asparagine residue at position 308 in YU2 (compared with histidine or arginine in envelopes from the other strains) or leucine at position 317 in YU2 (compared with phenylalanine in envelopes from the other strains). In contrast, PGT126 bound most weakly to JRFL-derived gp120 and gp140 and bound strongly to unmodified YU2 gp120. Because the core V3 sequence of JRFL Env is identical to that of Env from some other tested strains, the specificity of PGT126 binding must be determined by sequences outside this region or by differences in conformation or glycan structure of the different envelope proteins.

Antibody PG9, directed against variable loop peptide and glycan epitopes, bound weakly, but significantly, only to BG505 forms of gp120 and gp140. PG9 preferentially binds envelope trimers [61, 64] but is capable of binding monomers under certain conditions [11, 65, 66]. Binding of PG9 is reported to be strongly dependent on the presence of glycosylation at positions 156 and 160 [11, 64–67]. Among the constructs tested for Fig 4, all but QH0692 have an asparagine at position 160 that can potentially be glycosylated, but only the unmodified YU2 has a potential glycosylation site at position 156.

Two additional antibodies directed against protein and glycan determinants in variable loops failed to bind to any of the yeast-displayed envelopes. One of these was PGT121, which is reported to be directed against V3 region peptide and glycan, though with less specificity for high mannose at position 332 than for 10–1074 [60, 61]. Inefficient binding of PGT121 to endogenous yeast glycoproteins has been reported previously [63]. The second was PGT145, which is directed primarily against a quaternary epitope including a glycan component in the V1/V2 region. PGT145 recognizes oligomannose structures at position 160 of Env, but the particular configuration of the glycan and quaternary interactions appear to be important for the recognition [61, 68]. Binding of PGT145 is also strongly dependent on glycosylation at positions 156 (which can only occur in our unmodified YU2 constructs) and 160 (which can occur in all but the QH0692 constructs) [11, 64–67]. Binding of PGT145 and PG9 has been reported to be negatively affected by treatments enriching for high mannose glycan at recognition sites [66, 67], so yeast glycosylation patterns might similarly interfere with binding of these antibodies.

#### Binding of anti-MPER antibodies to yeast-displayed Env

Surface-expressed forms of gp140 Env bound antibodies directed against regions of the Membrane Proximal External Region (MPER) of gp41. No binding of these antibodies to yeast-displayed forms of gp120 was detected, despite reports that they may also recognize epitopes on gp120 [69]. The four tested anti-MPER antibodies all exhibited little or no binding to unmodified forms of gp140, but robust binding to modified dSOSIP and dsm forms of the envelope (Fig 3 and S2 Fig). Thus, although the stabilizing mutations were originally identified based on their abilities to enhance the binding of anti-CD4 binding site antibodies to Env, they also affect the gp41 moiety of the protein in ways that promote binding of antibodies directed against this region.

**Fig 7:**
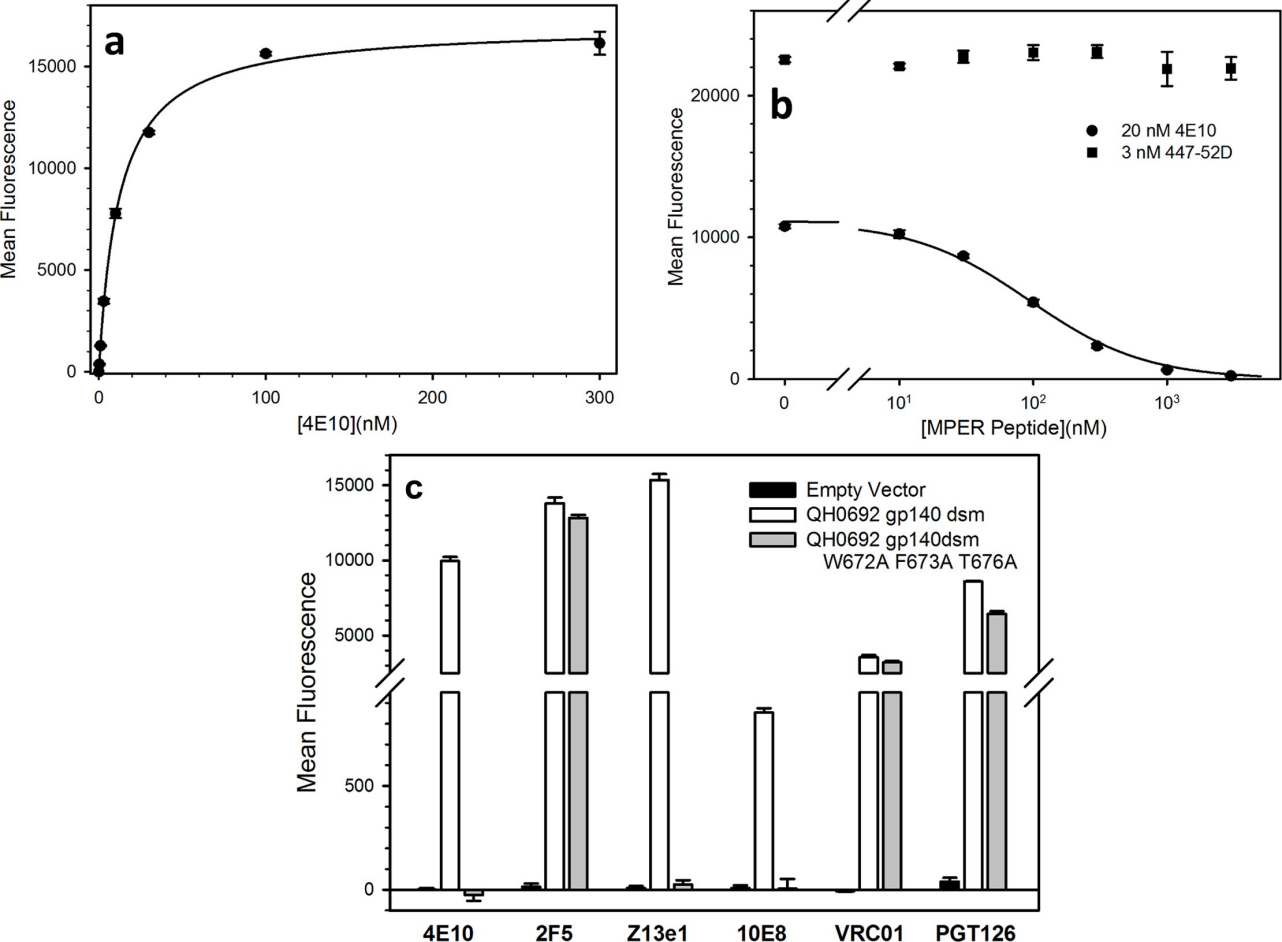
Binding of anti-MPER antibody 4E10 to gp140dsm from viral strain QH0692. (a) Saturation binding of 4E10 to QH0692 gp140dsm yields a K_d_ of 12 ± 1 nM. (b) Binding of antibody 4E10 (20 nM) to QH0692 gp140dsm can be competed effectively by 23-mer MPER peptide with a nanoMolar logIC_50_ of 2.0 ± 0.02, corresponding to a K_i_ of 37 nM for peptide binding to 4E10. (c) mutations to the 4E10 binding consensus sequence abolishes binding of MPER-directed antibodies 4E10, 10E8, and Z13e1 but has minimal effect on binding of 2F5, which recognizes a distinct gp41 epitope, or on binding of VRC01, directed against the CD4 binding site, or on binding of PGT126, directed against variable loop peptide and glycan. All antibodies were used at a concentration of 100 nM.

**1) 4E10.** The broadly neutralizing antibody 4E10 effectively bound stabilized forms of gp140 from all four tested viral strains (Figs 3 and 4 and S1 and S2 Tables). 4E10 recognizes the core sequence W^672^FX(I/L)(T/S)XX(L/I)W^680^(where X indicates residues that do not play major roles in recognition) [70, 71]. The important residues in this motif are preserved in all the Env constructs that we tested. Saturation binding experiments with 4E10 yielded a K_d_ value of 12 ± 1 nM to QH0692 gp140dsm (Fig 7A) and binding of 4E10 to the gp140dsm could be competed using a synthetic MPER peptide (sequence LELDKWASLWNWFDITNWLWYIK, corresponding to Env residues 661–683) with a logIC_50_ of 2.0 ± 0.02, corresponding to a K_i_ of 37 nM for binding of the peptide to 4E10. (Fig 7B). Also, mutation of three residues W672A, F673A, and T676A, which have each individually been shown to inhibit binding of 4E10 to its epitope [72–74] blocked binding of 4E10, 10E8, and Z13e1 to the QH0692 gp140dsm without significantly diminishing binding of 2F5 to its non-overlapping MPER epitope, or affecting binding of the anti-CD4 binding site antibody VRC01 or the anti-V3 loop antibody PGT126 (Fig 7C).

**Fig 8:**
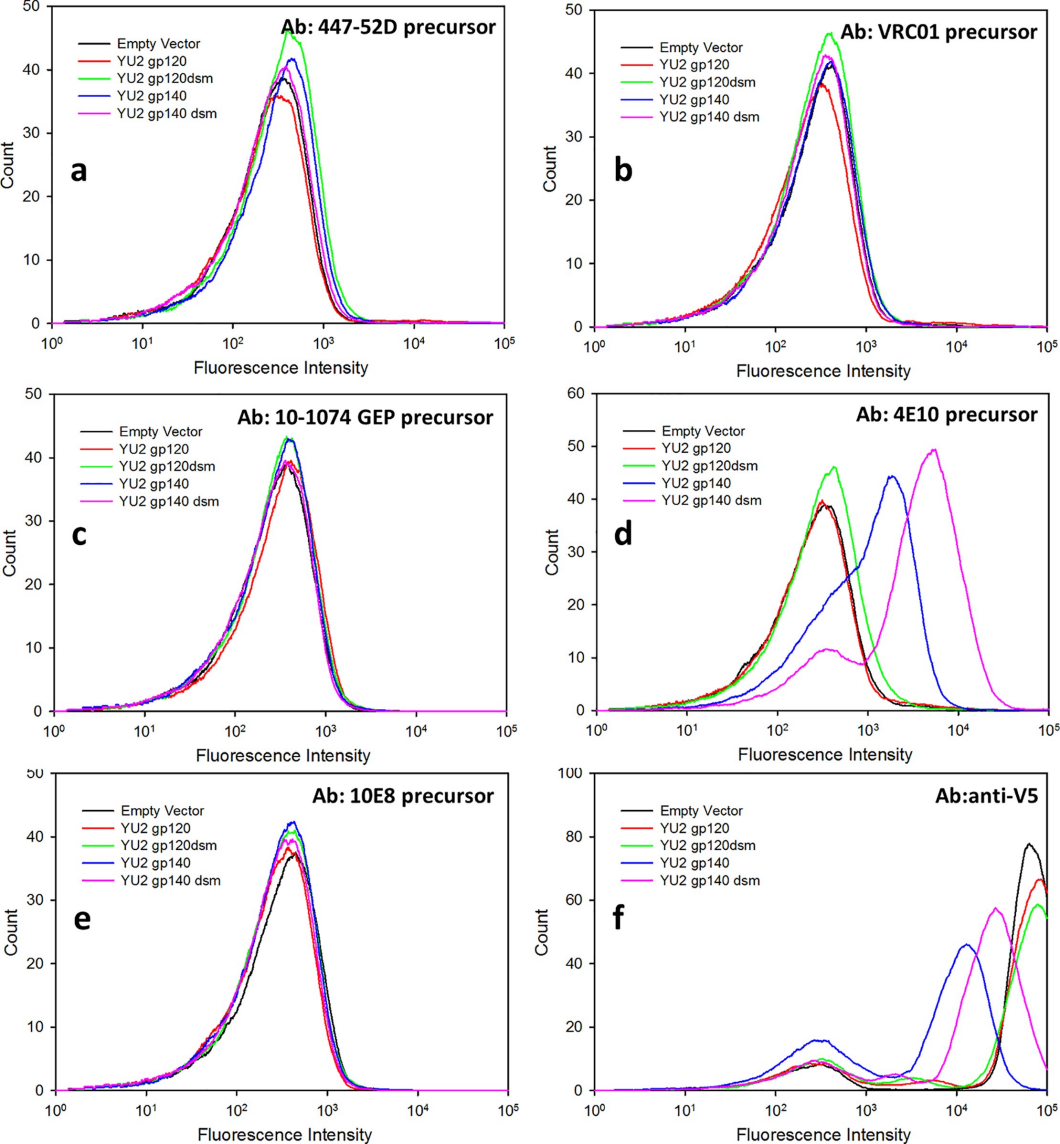
Binding of precursors of anti-Env antibodies to yeast-displayed Env. The figure shows a fluorescence analysis of ~10,000 cells following incubation with the indicated primary antibodies and fluorescent secondary antibody. Anti-Env antibodies were present at a concentration of 100nM. Anti-V5 antibody was present at a concentration of 20 nM. For each panel, the histograms shown are for cells with empty vector (black) or expressing YU2 gp120 (red) YU2 gp120dsm (green), YU2gp140 (blue), and YU2 gp140dsm (pink). Cells were incubated with (a) precursor for antibody 447-52D; (b) precursor for antibody VRC01; (c) precursor for antibody 10–1074; (d) precursor for antibody 4E10; (e) precursor for antibody 10E8, and (f) anti-V5 antibody.

**2) 10E8.** Anti-MPER antibody 10E8 bound particularly strongly to stabilized YU2 gp140dsm and more weakly to QH0692 gp140dsm than to Env from the other viral strains. 10E8 recognizes the sequence N^671^WFDITNWLWYIR^683^[75] residing at the extreme C-terminal of the Env gp140 constructs that we tested, partially overlapping the recognition sequences for Z13e1 and 4E10. The C-terminal arginine in this sequence has been implicated in recognition by 10E8, since replacement of Lys or Arg at position 683 reduces Env binding and pseudovirus neutralization by 10E8 [75]. This may explain why we observe efficient binding of 10E8 to YU2 Env constructs, which contain Lys at their C-terminal position 683 and poor binding to the QH0692 gp140dsm, which does not contain Lys at this position. However, this does not explain the binding seen to JRFL constructs, which is truncated at position 681. (Note that, as described in Materials and Methods fusion to Aga2p in vector pYD5 introduces a GluPhe sequence following the final amino acids of the Env constructs.) Binding of 10E8 to QH0692 gp140dsm was also blocked by introduction of the triple mutation W672A F673A T676A (Fig 8), consistent with previously reported effects of these individual substitutions on 10E8 binding [75].

**3) Z13e1.** Antibody Z13e1 bound effectively to all four tested forms of gp140dsm. Z13e1 recognizes the sequence W^666^ASLWNWFDITN^677^ [73], which is conserved in the tested Env constructs except for the substitution of Ser for Thr at position 676 in BG505 and the replacement of Asp667 by Lys in YU2 and by Arg in QH0692. However, serine at position 676 is found in viruses effectively neutralized by Z13e1 [76] and Ala substitutions at positions 676 and 677 do not inhibit the ability of Z13e1 to neutralize pseudotyped virus in single round infection assays [73].
**4) 2F5.** The anti-MPER antibody 2F5 recognizes the sequence L^661^ELDKWASL^669^ [74, 77–79], possibly with additional conformational determinants [80]. This core sequence is conserved in the yeast-displayed Env constructs except for the substitution of alanine for glutamic acid at position 662 in the YU2 and BG505 Env constructs. This substitution has previously been shown to have little effect on 2F5 binding [74]. Binding of 2F5 is not affected by the mutation W672A F673A T676A that blocks binding of other anti-MPER antibodies.
**5) CAP248.** This antibody, which did not bind to any form of yeast-expressed Env, is reported to recognize quaternary epitopes on both gp120 and gp41 that reside close to the viral membrane. CAP248 binding is affected by mutations at the furin cleavage site, and is independent of any single glycan modification on Env [81, 82]. Thus, recognition of yeast-displayed gp140 could be blocked by an altered Env conformation, by the alterations introduced into the furin cleavage site of the yeast-displayed gp140, by the lack of membranes and membrane-inserted sequences in the yeast display system, or blocking of epitopes by the large yeast glycan moieties.

### Binding of unmutated precursor forms of anti-Env antibodies to yeast-displayed Env

Since the inability of unmutated precursor forms of bnAbs to bind to Env is considered to be a major impediment to eliciting protective immune responses to HIV, a long-term goal of this project is to identify variant forms of Env with enhanced affinity for antibody precursors. Thus, we examined binding of yeast-displayed forms of Env to several reconstructed bnAb precursors of antibodies (Fig 8). In contrast to the robust binding seen for binding of mature forms of 447-52D, VRC01, and 10E8 to yeast-displayed Env, none of the precursor forms of these three antibodies bound to unmodified or dsm forms of YU2 gp120 or gp140. This is consistent with previous observations of the low affinity of binding of 447-52D [34], VRC01 [19, 83], and 10E8 [84] precursors to Env. The precursor form of 4E10 did, in fact, bind to yeast-displayed gp140 forms of YU2, however, the affinity for such binding was ~20-fold weaker than the affinity of mature 4E10 for the same constructs and the apparent number of binding sites for the precursor was ~3-fold lower than for the mature antibody (S3 Fig). Weak binding of 4E10 precursor to YU2 gp140 has been reported previously [85]. Binding of the 4E10 precursor to yeast-displayed Env from viral strain QH0692 was much lower than for YU2 (results not shown).

### Effects of Env glycosylation

Core N-glycosylation of proteins is similar in yeast and mammals, however, subsequent processing of glycans differs in the two organisms. Instead of undergoing processing to complex or high mannose forms, as in animal cells, secreted yeast glycoproteins undergo addition of an initial α-1,6-mannose residue catalyzed by the mannosyltransferase Och1p followed by addition of mannose residues to form hyperglycosylated structures [86]. We investigated the possibility of enhancing antigenic properties of yeast-displayed Env by deleting *OCH1* as well as the *MNN1* and *MNN4* genes involved in polymerization of large polyglycosylated chains at N-glycosylation sites [86, 87]. However, this combination of mutations resulted in poor cell growth under conditions used for Env induction and was not pursued further.

The antibody 2G12, which is reported to be directed primarily against high mannose glycan epitopes [88, 89], binds to yeast-displayed gp120 and gp140. 2G12 has previously been found to bind endogenous yeast glycoproteins in yeast strains deleted for *OCH1*, *MNN1*, and *MNN4* [90]. As shown in Fig 4, we detect a weak interaction between 2G12 and some unknown component on *OCH1*^+^ *MNN1*^+^ *MNN4*^+^ cells that do not express any Env. However, the levels of binding of 2G12 to yeast strains displaying various forms of Env were much greater than to cells containing empty vector alone. The lowest levels of 2G12 binding among the tested constructs were to the gp120 and gp140 forms of YU2 Env, most likely reflecting a particularly unfavorable pattern of N-glycosylation of YU2 Env compared to envelopes from other tested viral strains.

To examine effects of glycosylation on antibody binding to yeast-displayed Env, we treated yeast-displayed QH0692 gp120dsm and gp140dsm proteins with two glycosidases; endoglycosidase H (endoH), which cleaves between the N-acetylglucosamine residues of the chitobiose core of N-linked glycans, leaving one N-acetylglucosamine residue attached to the backbone asparagine [91] and Peptide:N-Glycosidase F (PNGase F), which cleaves the linkage between the backbone asparagine and the first N-acetylglucosamine residue [92]. (We also examined the effects of treating cells with tunicamycin, an inhibitor of N-linked glycosylation in cells. However, based both on antibody binding and immunoblotting with anti Env antibodies, tunicamycin treatment severely inhibited expression of Env-Aga2p constructs, and thus was not pursued further.)

**Fig 9:**
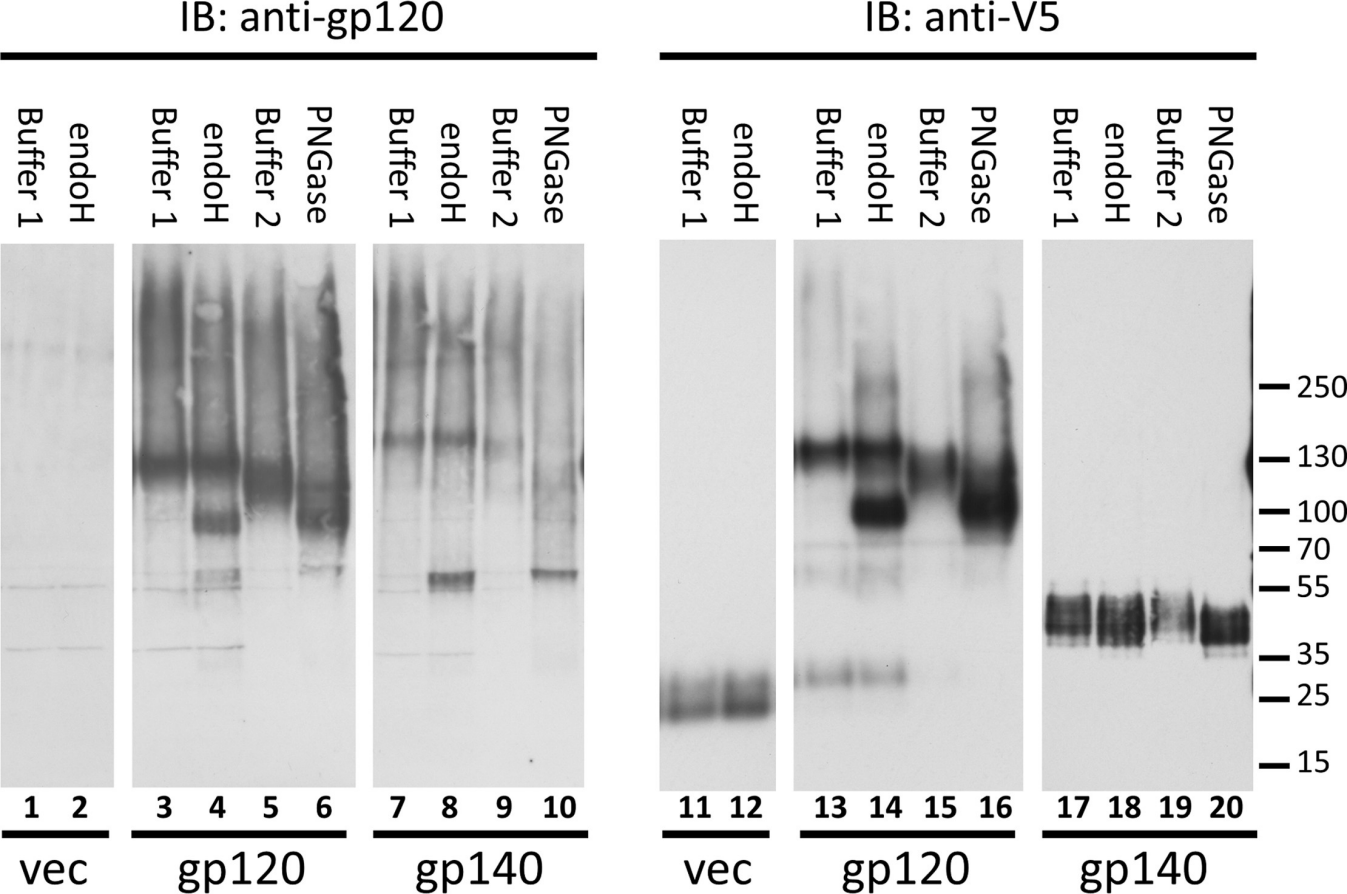
Immunoblot comparing effects of glycosidase treatments of yeast surface displayed QH0692 gp140dsm. All samples were released from yeast cells by treatment with DTT. Lanes 1–10 were probed with polyclonal anti-gp120 antibody. Lanes 11–20 were probed with anti-V5 epitope antibody. Lanes 1, 2, 11, and 12, cells containing empty vector (expressing V5-Aga2p but not Env). Lanes 3–6 and 13–16, cells expressing QH0692 gp120dsm-Aga2p. Lanes 7–10 and 17–20, cells expressing QH0692 gp140dsm-Aga2p. Cells in lanes 1, 3, 7, 11, 13, and 17 were incubated is Buffer 1 (see Materials and Methods) as controls. Cells in lanes 2, 4, 8, 12, 14, and 18 were incubated with endoH in Buffer 1. Cells in lanes 5, 9, 15, and 19 were incubated is Buffer 2 (see Materials and Methods) as controls. Lanes 6, 10, 16, and 20 were incubated with PNGase F in Buffer 2.

The effectiveness of cleavage by both endoH and PNGase F was determined from examination of the effects of enzyme treatments on immunoblots of the yeast expressed protein probed with an anti-gp120 polyclonal antibody and a monoclonal antibody recognizing the V5 epitope. As noted below, analysis of the patterns of bands on these immunoblots also allowed determination of the efficiencies of cleavage at the furin site in gp120 and gp140 species by the endogenous yeast Kex2p protease. Cleavage of glycans by the enzyme treatments was apparent from the detection of faster-migrating species and the depletion of high molecular weight species upon immunoblotting of protein released from intact cells by reducing conditions that disrupt the Aga1p-Aga2p interaction that anchors the constructs at the cell wall (Fig 9). Untreated gp120 and gp140 ran as diverse high molecular weight species with prominent bands at about 120 kDa and 140 kDa, respectively, on blots probed with the anti-gp120 antibody (Fig 9, lanes 3, 5, 7, and 9). Similar sets of bands are visible for the gp120 probed with anti-V5 antibody, as expected, since the V5 remains covalently attached to the gp120 constructs (Fig 9, lanes 13 and 15).

Glycosidase treatments of the gp120 construct resulted in the appearance of a predominant band or set of bands of about 100 kDa, detected by anti-gp120 and anti-V5 antibodies as seen in Fig 9 lanes 4, 6, 14, and 16. This is most likely comprised of partially deglycosylated gp120-Aga2p fusion protein, which has a predicted mass of 67 kDa for its protein component. Glycosidase treatments of gp140 result in the appearance of a band at ~70 kDa on blots probed with anti-gp120 (Fig 9, lanes 8 and 10), likely corresponding to deglycosylated gp120 (predicted mass of the protein component; 57kDa) derived from cleavage at the furin cleavage site between gp120 and the fused gp41-Aga2p moiety. As expected, probing of gp140 samples with anti-V5 antibody detects the cleaved gp41-Aga2p moiety (Fig 9, lanes 17–20) running slightly slower than the V5-Aga2p species expressed by vector with no inserted Env (Fig 9, lanes 11 and 12). The presence of the gp41-Aga2p species as essentially the only band on the anti-V5 blot of the yeast-displayed gp140 indicates that cleavage between gp120 and gp41 by the endogenous yeast Kex2p protease is efficient for the tested constructs.

While mutation of the furin cleavage site at the C-terminal of gp120 to maintain anchoring to Aga2p appears to be generally effective in blocking cleavage, a small residual amount of cleavage at or near this site seems to persist, based on: 1) detection of a minor population of a low molecular weight species by anti-V5 antibody in samples derived from gp120-expressing cells (Fig 9, lanes 13–14). This species runs slightly slower than the V5-Aga2p moiety present in the empty vector (Fig 9, lanes 11–12) and faster than the species corresponding to gp41-Aga2p (Fig 9, lanes 17–20). It is most evident in samples incubated in the buffer for endoH (Buffer 1; compare lanes 13 and 14 with lanes 15 and 16); 2) detection by anti-gp120 antibody of a minor band in glycosidase-treated gp120-expressing samples (Fig 9, lanes 4 and 6) that has the same electrophoretic mobility as the predominant deglycosylated gp120 band seen in samples derived from gp140-expressing cells (Fig 9, lanes 8 and 10). This band is also most pronounced in samples incubated in buffer for endoH.

**Fig 10:**
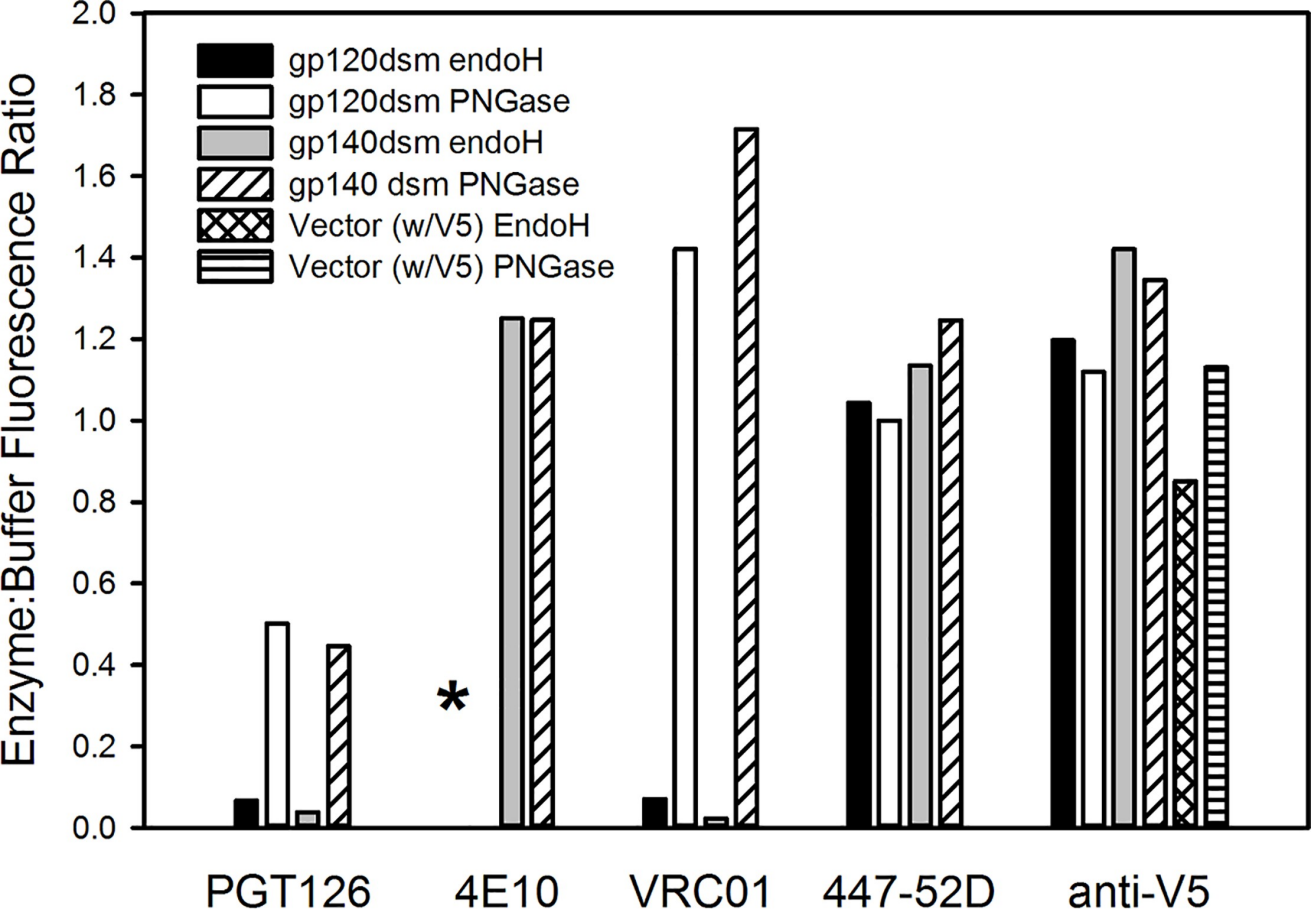
Treatment of yeast surface displayed QH0692 gp120dsm and gp140dsm with glycosidases affects the binding of some anti-HIV bnAbs. For each tested antibody, binding is indicated as the ratio of the mean fluorescence resulting from binding to glycosidase-treated cells to the fluorescence resulting from binding to cells treated only with the glycosidase buffer control. The anti-envelope and anti-V5 antibodies were used at concentrations of 50 nM and 20 nM, respectively. Digestions with endo H and PNGase F were performed in Buffer 1 and Buffer 2, respectively (See Materials and methods). The asterisk indicates that strains containing Env gp120 did not bind to 4E10. The results shown are averages from two independent experiments.

Enzymatic deglycosylation of yeast-displayed Env had varying effects on the binding of different antibodies to yeast-displayed constructs (Fig 10). Treatment with endoH reduced PGT126 binding to both gp120 and gp140 constructs to less than 10% of the level observed for untreated cells (p < 0.05) and resulted in similar reductions in VRC01 binding (p < 0.02). On the other hand, treatment with PNGase F reduced PGT126 binding to a lesser, and less significant, extent and actually appeared to result in a slight enhancement of VRC01 binding. VRC01 is not generally considered to be a glycan dependent antibody, but it has been reported to contact the N-acetylglucosamine linked to Asn276 [93], which is conserved in all the tested yeast-displayed constructs. However, this does not explain the loss of VRC01 binding upon endoH treatment, since endoH leaves the initial N-acetylglucosamine attached to asparagine. Neutralization of HIV by VRC01 has also been observed to be enhanced by treatment of cells with kifunensin, which inhibits formation of complex N-linked glycans [94]. Thus, certain glycan modifications may block accessibility of VRC01 to the CD4 binding site. Glycan removal had little effect on the binding of the anti-V3 loop antibody 447-52D to gp120 or gp140, on the binding of the anti-MPER antibody 4E10 to gp140, or on the binding of anti-V5 antibodies to gp120, gp140, and vector expressing the epitope without any Env (Fig 10).

### Comparison with yeast-displayed gp140 expressed in mammalian cells

**Fig 11:**
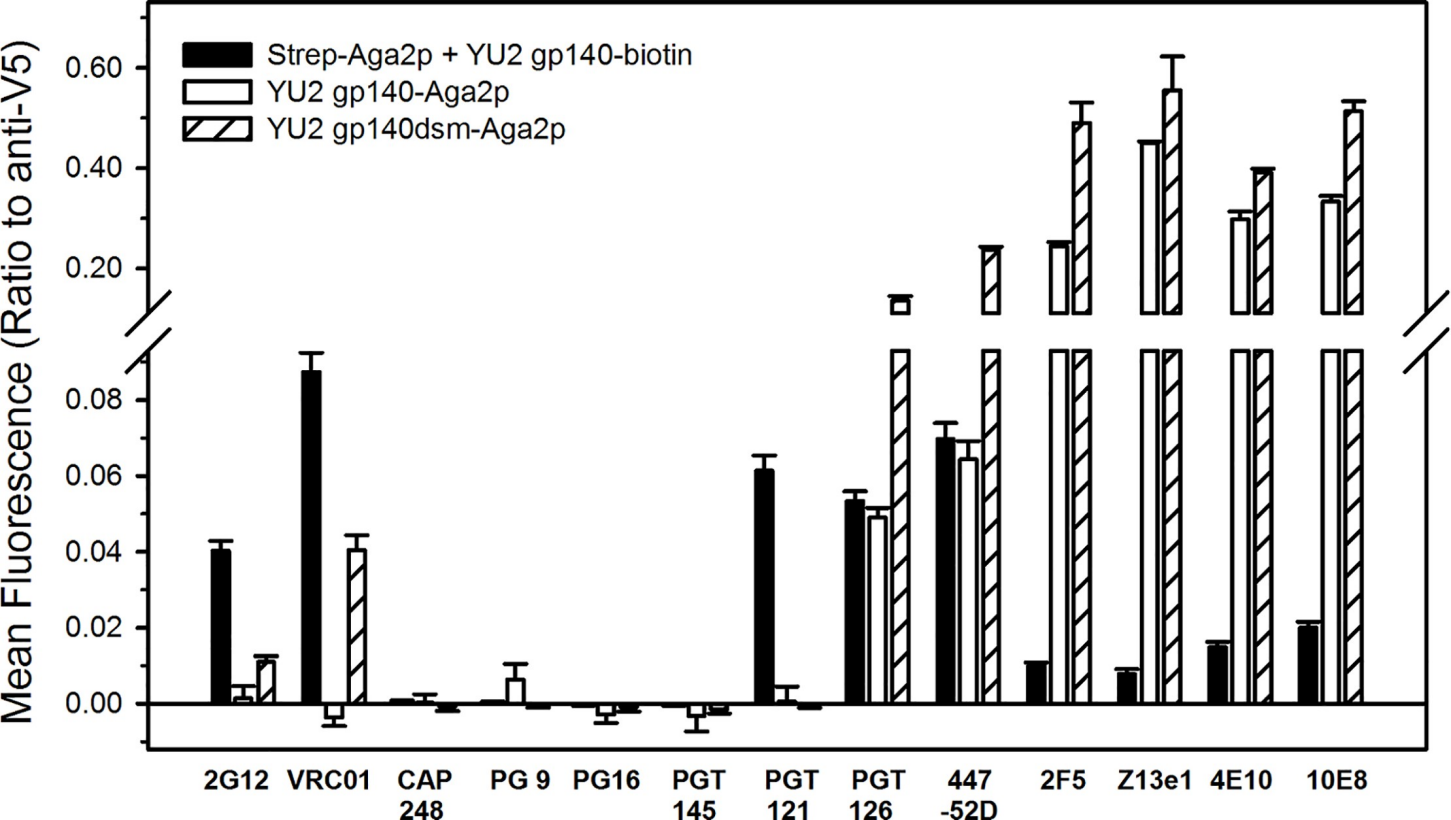
Comparison between antibody binding to yeast surface displayed gp140 and gp140 purified from mammalian cells. Unmodified YU2 gp140 and YU2 gp140dsm, containing stabilizing mutations (see text), were expressed as a fusions at their C-termini to Aga2p. Alternatively, purified, biotinylated YU2 gp140 (17 nM) was bound (at a saturating concentration) to yeast cells displaying a streptavidin-Aga2p fusion protein. Binding was detected by flow cytometry following incubation with fluorescently tagged secondary antibodies (Antibody concentrations were: 70 nM (anti-Env antibodies); 20 nM (anti-V5 tag antibody). The indicated values are the ratios of the mean fluorescence intensities for the relevant anti-HIV antibodies to the mean fluorescence intensity for binding of anti-V5 epitope antibody to each yeast strain (measured in the absence of purified biotinylated gp140). Fluorescence intensity for binding of anti-V5 epitope antibodies to the streptavidin-Aga2p fusion (in the absence of purified biotinylated gp140) was 7.5 (± 0.4, n = 6)-fold higher level than the level for binding of anti-V5 to yeast-displayed YU2 gp140 fused to Aga2p and 2.5 (± 0.1, n = 6)-fold higher level for anti-V5 binding to YU2 gp140dsm fused to Aga2p (data not shown). Secondary antibody incubation was performed at 4°C and flow cytometry was performed with cells kept on ice.

As a reference for determining the efficiency of display of different forms of Env in the yeast system, we used an alternate display system in which a yeast strain displaying streptavidin at the cell surface was pre-bound to a saturating concentration of biotinylated, trimeric, cleavage-deficient YU2 gp140 purified following expression in HEK293 cells [95] (Fig 11). Based on binding of anti-V5 epitope antibodies, the streptavidin-Aga2p fusion was expressed at a 7.5 (± 0.4, n = 6)-fold higher level than the yeast-expressed fusion of a gp140 from viral strain YU2 to Aga2p and a 2.5 (± 0.1, n = 6)-fold higher level than the yeast-expressed stabilized YU2 gp140dsm fused to Aga2p (data not shown). Nonetheless, various antibodies, including all tested anti-gp41 antibodies and two anti-V3 loop antibodies (447-52D and PGT126) displayed higher levels of binding to the gp140dsm-Aga2p construct than to the purified gp140 bound to displayed streptavidin, particularly when binding was normalized for expression levels based on anti-V5 binding (Fig 10). Only low levels of binding of anti-MPER antibodies to the gp140 from mammalian cells were detected, indicating that gp41 of the yeast-expressed Env is more accessible than is the case for the protein from mammalian cells. This may, in part, reflect the fact that the mammalian cell-expressed gp140 lacked an effective furin cleavage site [95, 96]. Two anti-V3 loop antibodies, 447-52D and PGT126, bound similarly to the mammalian- and yeast-expressed unmodified forms of YU2 gp140, but exhibited enhanced binding to the stabilized gp140dsm form (p < 0.01 for each). Three of the tested antibodies (2G12, VRC01, PGT121), bound better to the mammalian cell-expressed protein than to the yeast- expressed envelopes (p <0.002 for each). For two of these (2G12 and PGT121) that are at least partially glycan specific, this may reflect an inability to recognize yeast glycans. Four antibodies (CAP248, PG9, PG16, and PGT145) failed to exhibit significant binding to any form of gp140 displayed on yeast.

## Conclusions

As a step towards using large-scale random mutagenesis in the yeast surface display system for the development of immunogens with new binding specificities for anti-HIV antibodies, we characterized antibody recognition of different forms of HIV Env displayed on the surface of yeast cells. Anti-HIV antibodies bound yeast-displayed envelope constructs that were fused either directly to the cell wall protein Sag1p or to the protein Aga2p, which binds the cell wall protein Aga1p. Fusion of the C-termini of envelope proteins to Aga2p provided the highest levels of expression and antibody binding. Gp140-Aga2p fusions containing a furin cleavage site optimized for recognition by the yeast furin homolog, Kex2p, underwent efficient cleavage in the yeast display system. Mutation of the furin cleavage site to block cleavage of gp120-Aga2p fusion constructs (to maintain surface display) was nearly completely effective.

We find that amino acid substitutions identified in a previous screen for variant forms of JRFL-derived gp140 that enhance binding of the CD4-binding site-directed antibody VRC01 [22] also enhance the binding of additional antibodies with diverse specificities. Furthermore, the enhancements in antibody binding were maintained when the corresponding substitutions were introduced into forms of gp120 and gp140 derived from other viral strains. The improvements in antibody binding conferred by these mutations are likely to arise, at least in part, from increased expression or stability of the mutant forms of Env, since they exhibited higher levels of binding of anti-V5 epitope and polyclonal anti-gp120 antibodies compared with the corresponding dSOSIP forms.

Binding to yeast-displayed forms of Env was detected for antibodies against several known targets of bNAbs, including classes reported to primarily recognize linear epitopes (447-52D, 2219, 4E10, and 10E8), classes recognizing glycan or combined glycan/protein epitopes (2G12, PGT126 and 10–1074), and a conformation-specific antibody (VRC01). Whereas previous studies of gp120 in the yeast display system focused primarily on a core form of the envelope lacking loop regions [19–22], precluding recognition by anti-loop antibodies, we found that full-length mutated versions of gp120, as well as gp140, bound to antibodies specific for the variable loops. The specificities and affinities of recognition of yeast-displayed Env by several classes of anti-Env antibodies were confirmed by saturation binding assays and by competitive binding studies using synthetic peptides, gp120 purified from mammalian cells, and a soluble form of the CD4 receptor (for the anti-CD4 binding site antibody VRC01). Furthermore, binding of anti-CD4 binding site and anti-MPER antibodies was blocked by mutations in regions of Env known to be involved in antibody recognition.

Despite differences between the processing of N-linked glycosylation in yeast and mammals, antibodies PGT126, 10–1074, and 2G12 that are at least partially specific for glycan, bound to yeast-expressed and displayed forms of Env. Treatment of yeast cells displaying Env with glycosidase diminished binding of PGT126. We were also surprised to note that treatment of Env-expressing cells with endoH also diminished binding of VRC01, consistent with a role for glycan in recognition by this antibody. Collectively, the effects of glycosidases and the observed specificities of antibody binding were generally consistent with the observation that patterns of N-linked glycosylation in yeast are more similar to the high mannose structures seen in mammalian cells than they are to mammalian complex type glycosylation.

Levels of antibody binding to gp140 expressed in, and displayed on, yeast cells were comparable to those that can be obtained by binding biotinylated gp140 purified from mammalian cells to yeast displaying streptavidin at the cell surface, indicating that yeast can efficiently express and process Env to a form with authentic antigenic properties. While some antibodies that are known to recognize glycan, such as 2G12 and PGT121, recognized the purified mammalian-derived gp140 better than the yeast-expressed version, others, such as anti-MPER antibodies and PGT126, which is also believed to recognize glycan, exhibited higher levels of binding to the yeast-expressed Env. This makes yeast display an attractive system for developing immunogens for certain bnAb responses, particularly those for MPER-specific antibodies. However, some anti-Env antibodies (CAP248, PG9, PG16, and PGT145) did not bind to either the yeast-expressed or mammalian-derived forms of Env displayed on yeast. Since epitopes for all of these antibodies are reported to involve some component of quaternary structure of Env, this may reflect some non-native structural aspects of both the yeast-displayed and purified mammalian cell-expressed forms of Env.

The results presented here indicate that, despite distinct differences between yeast-displayed envelope and the protein’s native state on viral membranes, the yeast system can be used for surface display of envelope that maintains high affinity for a variety of human anti-Env antibodies, including some with potent broadly neutralizing properties. Consistent with diverse reports showing low binding affinity of unmutated precursors of broadly neutralizing antibodies for Env, out of five predicted precursors to bnAbs that were tested, only the precursor for 4E10 displayed some affinity for yeast-expressed YU2 gp140 and the binding affinity of this precursor was considerably weaker than the affinity of mature 4E10 for the same constructs. The poor binding that we observe between bnAb precursors and Env, allows use of this system to develop mutated forms of the viral protein with enhanced affinities for the precursors- an effort that is currently underway for the generation of immunogens capable of eliciting broadly neutralizing responses.

## Materials and methods

### Materials

The following anti-HIV envelope antibodies and additional reagents were obtained from the NIH AIDS Reagents Program Division of AIDS, NIAID, NIH (original providers indicated in parentheses): 10–1074 (Dr. Michel C. Nussenzweig), 2219, 2191, 268-D (Dr. Susan Zolla-Pazner), 10E8 (Dr. Mark Connors), 17b, 39F (Dr. James E. Robinson), 2F5, 2G12 (Polymun Scientific), 4E10, MPER peptide, purified Bal gp120 (NIAID), F425B4e8 (Dr. Marshall Posner and Dr. Lisa Cavacini), PG9, PG16, PGT121, PGT126, PGT145 (International AIDS Vaccine Initiative), Z13e1 (Dr. Michael Zwick). Antibody 447-52D was expressed from a synthetic gene in HEK293 cells and purified essentially as described [97]. CAP248-2B was a gift from the laboratory of Dr. Peter Kwong. 1069-D6, a human anti-HPV antibody used as a negative control in this study, was a gift from Dr. James Kobie. The mouse anti-V5 epitope antibody was from Pierce (ThermoFisher) and the goat anti-gp120 polyclonal antibody was from Meridian Bioscience. The plasmid vector pYD1 was a gift from the laboratory of Dr. Shohei Koide and vector pYD5 was purchased from Transplantation Biology Research Center, Boston, MA. Yeast transformations were performed using the Frozen-EZ Yeast kit from Zymo Research. Plasmid minipreps from E.coli were done using the SV Plus Miniprep kit from Promega. Restriction enzymes and other enzymes required for molecular biology techniques, as well as Phusion high-fidelity polymerase and glycosidases were purchased from New England Biolabs. Fluorescent antibodies were purchased from ThermoFisher Scientific. HRP-conjugated secondary antibodies and polyacrylamide gels were from Bio-Rad.

Reconstructed predicted precursor forms of antibodies 10E8 [98]and 10–1074 [60] were obtained, from Dr. Peter Kwong of NIAID and Dr. Nussenzweig, of Rockefeller University, respectively. Precursor forms of VRC01, 447-52D, and 4E10 were reconstructed using the IgBLAST tool ((http://www.ncbi.nlm.nih.gov/igblast/)). V(D)J segments with the highest identity to the mature antibody sequence were used. Because of uncertainty in reconstructing VDJ joints, which form the HCDR3 regions, the mature antibody HCDR3 sequence was maintained in each case. Sequences of relevant portions of the reconstructed precursors for 447-52D, VRC01, and 4E10 are shown in S4 Fig. Cloning, transfection of HEK293T cells, and purification of reconstructed precursor antibodies were performed as described previously [97].

### Yeast strains and plasmids

The host yeast strains used in this study are: BCY123 (*Mat****a*** *pep4*::*HIS3 prb1*::*LEU2 bar1*::HisG *lys2*::*GAL1/10*-*GAL4 can1 ade2 trp1 ura3 his3 leu2-3*, *112*)[99] and EBY100 (*MAT***a** *AGA1*::*GAL1*-*AGA1*::*URA3 ura3-52 trp1 leu2-delta200 his3-delta200 pep4*::*HIS3 prbd1*.*6R can1 GAL*), which overexpresses Aga1p under the *GAL1* promoter [29].

The yeast strains, plasmids and oligonucleotides used in this study are listed in S3 Table together with the specifics of plasmid construction. Env and streptavidin genes were synthesized as yeast codon-optimized gBlocks (Integrated DNA Technologies) or genes (Genescript or Genewiz) based on algorithms available on the suppliers’ web sites.

To create Env-Aga2p fusions, the envelope genes (minus the native signal peptide) were amplified by PCR using Phusion high-fidelity polymerase and primers that incorporated appropriate restriction sites at the 5’ and 3’ ends of the PCR products (NheI and EcoR1 for pYD5 and HindIII and BstBI for pYD1). The restriction enzyme-digested genes were then ligated into similarly-digested pYD1[100] or pYD5[31] plasmid vectors. The expression of both constructs was under the control of the *GAL1* promoter. For the C-terminal Aga2p fusions in vector pYD5, the sequence AlaGly was added at the C-terminal of the Aga2p signal peptide to optimize cleavage of the signal peptide [31]. Insertion into pYD5 via the 3’ EcoRI site introduces the sequence GluPhe immediately following the inserted form of Env. For C-terminal fusions of Aga2p to gp120 constructs, furin cleavage was prevented in order to maintain surface anchoring. This was accomplished by mutating Arg residues at the C-terminal of the protein to Ser (R503S, R504S, R508S, and, where present, R511S) by incorporating the required changes into the 3’ PCR primer during gene amplification. Gp120 constructs were fused to Aga2p following residue 511 (HXB2 numbering)

Plasmid pMD1918, allowing expression of Env constructs fused at their C-termini to Sag1p, was constructed by subcloning the HindIII-EcoRI fragment containing the α-factor secretion signal from pPICZαA (Invitrogen Life Technologies/ Thermo Fisher Scientific) as a three-way ligation in combination with the ~1 Kb portion of the *SAG1* gene encoding a C-terminal fragment beginning at residue 332[101]. The *SAG1* fragment was PCR amplified using primers that added an EcoR1 site at the 5’ end and an XbaI site just after the termination codon. The HindIII-EcoR1 fragment encoding the secretion signal and the EcoRI-XbaI fragment containing the fragment of *SAG1* were ligated into HindIII-XbaI digested pYES2 (ThermoFisher Scientific). To insert Env constructs into pMD1918, the Env genes (minus the native signal peptide region) were amplified by PCR using Phusion high-fidelity polymerase with a 5’ primer that introduces an XhoI site, as well as 12 bases from the 3’ region of the alpha factor secretion signal and a V5 epitope tag, and a 3’ primer that introduces a SphI site. The XhoI-SphI-digested PCR product was ligated into similarly-cut pMD1918, placing the fusion construct under control of the *GAL1* promoter.

A yeast codon optimized gene encoding amino acids 8–139 of streptavidin from *Streptomyces avidinii* was purchased and amplified by PCR using primers that incorporated a 5’ NheI site and a 3’ EcoRI site. A Streptavidin-Aga2p fusion was constructed by subcloning the NheI/EcoRI-digested PCR product into pYD5 in a similar manner as the Env-Aga2p constructs.

Site-directed mutagenesis was performed as described previously [102, 103]). The oligonucleotides and templates used are listed in S3 Table.

### Assays of antibody binding to yeast-displayed Env

Cells of the appropriate strains were inoculated from solid media into unbuffered synthetic complete medium lacking tryptophan (for Aga2p fusions) or uracil (for Sag1p fusions) with raffinose as the sole carbon source. After overnight growth at 30°C, the cultures were diluted 10-60-fold in the same media buffered to pH 6 with 0.1 M sodium phosphate. These cultures were grown at 30°C for about 18 hours then were diluted again to an OD_600_ of 0.1–0.2. Expression was induced by the addition of galactose to a final concentration of 2% followed by culturing for 23–25 hours at 20°C. The cells were then pelleted, washed with PBI (phosphate buffered saline (pH 7.2) containing 1% BSA and protease inhibitors (EDTA-free protease inhibitor tablets by Roche or Pierce, prepared as directed by the manufacturer)) and used for binding assays either immediately or after brief storage at 4°C.

To measure antibody binding to cells, 0.5–1 × 10^6^ cells were incubated at 4°C with anti-Env or anti-V5 epitope antibodies in a total volume of 20 μl for 16–18 hours in PBI. The primary antibody concentrations used are indicated in the relevant figure legends. Concentrations of antibodies were chosen so as to be high enough to drive binding for interactions with dissociation constants as high as tens of nM, without causing antibody aggregation, depletion of secondary antibodies, or high levels of nonspecific binding (based on binding to empty vector and compared to the binding of nonspecific control antibodies). Cells were then washed with ice-cold PBS containing 1% BSA and then incubated with 25–50 nM Dylight_550_-, Alexa_633_- or Alexa_647_-conjugated secondary antibodies in PBI at 4°C or room temperature for 1 hour. Cells were then pelleted, resuspended in 0.4 ml ice-cold PBS and kept on ice or at room temperature while conducting flow cytometry with a 12- or 18-color LSRII (Becton Dickinson) using either a 532 nm laser with detection at 563–587 nm or a 633 nm laser and a detection channel of 640–680 nm. Data from flow cytometry was analyzed using the programs FlowJo and FCS Express. Histograms in figures were generated using 50% smoothing in FCS express. Competition of antibody binding by 23-mer MPER peptide and purified Bal gp120 was conducted by mixing the competitor with the galactose-induced cells just prior to incubation with the primary antibody.

Saturation and competitive binding data were analyzed using the nonlinear least squares functions of Sigmaplot. Evaluations of the significance of differences in pairwise comparisons of mean fluorescence levels from flow cytometry were conducted using two-tailed t-tests of fluorescence values that were logarithmically transformed to equalize variances. Statistical evaluations of fluorescence values that were normalized for expression levels were conducted by t-test without the transformation. Determination of whether fluorescence ratios differed from a value of 1 were conducted using one-sample t-tests. Statistical tests were performed using Sigmaplot.

To assay antibody binding on the yeast cell surface to biotinylated HIV Envelope expressed in HEK293F cells, 0.5–1 × 10^6^ yeast cells expressing the streptavidin-Aga2p fusion were induced as described above, washed with PBS containing 1% BSA and incubated with 17 nM YU2 gp140-biotin [95] at room temperature for 2 hours. Excess YU2 gp140-biotin was removed by pelleting and washing with PBS containing 1% BSA before antibody binding in PBI, as described above.

### Deglycosylation of yeast surface-displayed Env

Glycosidase treatment of yeast displayed envelope proteins was conducted by incubating 6–8 × 10^5^ galactose-induced cells with endoH or PNGase F (New England Biolabs), at a concentration of 20 U/μl, in the manufacturer-recommended buffers (Buffer 1, used with endo H, is 50 mM Sodium Citrate (pH 5.5) and Buffer 2, used with PNGase F, is composed of 50 mM Sodium Phosphate (pH 7.5) and 1% NP-40) at 37°C for 2 hours. The cells were then pelleted and washed with PBS containing 1% BSA before antibody binding and flow cytometry, as described above.

For immunoblot analysis of the glycosylated and deglycosylated samples, gel loading buffer (40 mM Tris-Cl (pH 6.8), 0.1 mM EDTA, 9M urea, 5% SDS, 100 mM DTT, 0.02 mg/ml bromphenol blue) was added to one fifth of the glycosidase-treated and control cells, after pelleting. Samples were electrophoresed on a Criterion 4–15% gradient gel, transferred to a 0.2 μM nitrocellulose membrane and blocked in PBS containing 5% non-fat milk for 2 hours. The membrane was then incubated with 1.8 μg/ml anti-gp120 polyclonal or 0.1 μg/ml anti-V5 epitope antibodies in PBS containing 3% BSA and 0.1% Tween-20 for 2 hours, washed thoroughly, and then incubated with appropriate HRP-conjugated secondary antibodies, diluted 10,000-fold in PBS containing 5% non-fat milk and 0.1% Tween-20, for 2 hours before visualizing by enhanced chemiluminescence using Supersignal West Dura Extended Duration Substrate, following the manufacturer-recommended protocol. All incubations for immunoblotting were performed at room temperature.

## Supporting information

S1 Fig Alignment of Env amino acid sequences used in this work. N-terminal signal sequences have been removed, since, except as noted in the text, these were replaced by the signal sequence from Aga2p or the α-factor secretion signal. The hydrophilic fusion peptide sequence (highlighted in green) and the optimized Kex2p-cleavage site (highlighted in purple) are as described [22]. Additional “stabilizing” mutations as described by Grimm et al., are highlighted in cyan. “SOSIP” mutations [104] are highlighted in grey. Note that the “unmodified” JRFL* and QH0692* sequences contain substitutions of the hydrophilic fusion peptide sequence described by Grimm et al., [22]. The sequence of Env from strain HXB2 (NCBI AAB50262.1) is shown for reference to standard numbering starting from the first codon in the signal sequence of HXB2 (numbers shown in bold and underlined). gp120 constructs were fused to Aga2p following residue 511 (HXB2 numbering). Other sequences are numbered with reference to the first codon after the Aga2p signal sequence in the yeast expression constructs. Alignment was performed using Clustal Omega [105]. Asterisks indicate positions where all sequences are identical, colons indicate strong conservation, periods indicate weak conservation.
(PDF)

S2 Fig Modified versions of yeast surface displayed gp140 from viral strains YU2, JRFL, and BG505 bind anti-HIV antibodies more efficiently than unmodified forms. The figure shows a flow cytometry histogram of fluorescence of ~10,000 cells following incubation with the indicated primary antibodies and fluorescent secondary antibody. Panels (a)-(f) compared the unmodified and dsm forms of YU2 gp140. Panels (g)-(k) compared the unmodified and dsm forms of JRFL gp140. Panels (l)-(o) compared the unmodified, SOSIP (but without the dSOSIP mutations), and dsm forms of BG505 gp140. (a), (g), (l) were probed with anti-gp120 polyclonal antibody (~66 nM); (b), (h), and (m)) were probed with anti-V3 loop antibody 2219 (320 nM); (c) and (i) were probed with anti-V3 loop antibody 447-52D (90 nM); (d), (j), and (n) were probed with anti-CD4 binding site antibody VRC01 (340 nM); (e), (k), and (o) were probed with anti-MPER region antibody 4E10 (200 nM); (f) was probed with anti-MPER region antibody 10E8 (70 nM). (Secondary antibody incubations and flow cytometry were performed at room temperature).
(PDF)

S3 Fig Saturation analysis of binding of predicted precursor and mature forms of 4E10 to YU2 gp120 and gp140. The indicated concentrations of antibodies were incubated with cells expressing different forms of Env as described in Materials and Methods. The binding assays were performed over different ranges of antibody concentrations for mature vs. precursor forms of 4E10 to facilitate fitting of binding curves in view of large differences in affinity. Error bars are smaller than the symbols. Based on triplicate biological replicates, the K_d_ for 4E10 mature binding to YU2 gp140dsm is 9.3 ± 0.4 and the relative B_max_ is 29,300 ± 300. For 4E10 precursor binding to YU2 gp140dsm the K_d_ is 163 ± 8 nM and the relative B_max_ is 9,300 ± 300.
(PDF)

S4 Fig Sequences of variable regions of reconstructed predicted precursors to antibodies 447-52D, VRC01, and 4E10. (PDF)

S1 Table Mean fluorescence values used to determine the average values for Fig 4. For antibodies tested with an n of 2, both values are indicated. If the n is greater than 2, average values with SEMs are indicated. If the mean fluorescence after subtraction of the mean fluorescence of secondary plus cells was negative, it was assigned a value of zero.
(PDF)

S2 Table Calculated p values for binding data presented in Fig 4. For each tested antibody a one-way ANOVA was performed followed by Dunnett’s post-test of the p value for deciding whether mean fluorescence intensities for each of the indicated viral strains are significantly different from the fluorescence values measured for empty vector tested with the same antibody. Because of the extreme differences in variance between measurements at low vs. high fluorescence values, the analysis of significance was performed on fluorescence values transformed by logarithmic transformation to equalize variances, after addition of a value of 200 to each fluorescence value (to accommodate negative fluorescence values following background subtraction of samples lacking primary antibody). The analysis was performed using Graphpad Prism software. Cells with grey shading indicate p values less than 0.05.
(PDF)

S3 Table Oligonucleotides and procedures for construction of different Env-expressing plasmids. (PDF)
