# Supplementary material for: Display of the HIV envelope protein at the yeast cell surface for immunogen development
Source: PLoS One. 2018 Oct 18;13(10):e0205756. doi: 10.1371/journal.pone.0205756 (PMC6193675; doi:10.1371/journal.pone.0205756)
Supplement: S1 Fig — N-terminal signal sequences have been removed, since, except as noted in the text, these were replaced by the signal sequence from Aga2p or the α-factor secretion signal. The hydrophilic fusion peptide sequence (highlighted in green) and the optimized Kex2p-cleavage site (highlighted in purple) are as described [22]. Additional “stabilizing” mutations as described by Grimm et al., are highlighted in cyan. “SOSIP” mutations [104] are highlighted in grey. Note that the “unmodified” JRFL* and QH0692* sequences contain substitutions of the hydrophilic fusion peptide sequence described by Grimm et al., [22]. The sequence of Env from strain HXB2 (NCBI AAB50262.1) is shown for reference to standard numbering starting from the first codon in the signal sequence of HXB2 (numbers shown in bold and underlined). gp120 constructs were fused to Aga2p following residue 511 (HXB2 numbering). Other sequences are numbered with reference to the first codon after the Aga2p signal sequence in the yeast expression constructs. Alignment was performed using Clustal Omega [105]. Asterisks indicate positions where all sequences are identical, colons indicate strong conservation, periods indicate weak conservation. (PDF) [file pone.0205756.s001.pdf]

Mathew *et al.*, S1 Fig.

```
YU2*          AEQLWVTVYYGVPVWKEATTTLFCASDAKAYDTEVHNVWATHACVPTDPNPQEVKLENVT   60
YU2dsm        AEQLWVTVYYGVPVWKEATTTLFCASDAKAYDTEVQNVWATHACVPTDPNPQEVKLENVT   60
JRFL*         ---LWVTVYYGVPVWKEATTTLFCASDAKAYDTEVHNVWATHACVPTDPNPQEVVLENVT   57
JRFLdsm       ---LWVTVYYGVPVWKEATTTLFCASDAKAYDTEVQNVWATHACVPTDPNPQEVVLENVT   57
BG505*        AENLWVTVYYGVPVWKDAETTLFCASDAKAYETEKHNVWATHACVPTDPNPQEIHLENVT   60
BG505SOSIP    AENLWVTVYYGVPVWKDAETTLFCASDAKAYETEKHNVWATHACVPTDPNPQEIHLENVT   60
BG505dsm      AENLWVTVYYGVPVWKDAETTLFCASDAKAYETEKQNVWATHACVPTDPNPQEIHLENVT   60
QH0692*       AENLWVTVYYGVPVWKEATTTLFCASDAKAYETEKHNVWATHACVPTDPNPQEVVLGNVT   60
QH0692dSOSIP  AENLWVTVYYGVPVWKEATTTLFCASDAKAYETEKHNVWATHACVPTDPNPQEVVLGNVT   60
QH0692dsm     AENLWVTVYYGVPVWKEATTTLFCASDAKAYETEKQNVWATHACVPTDPNPQEVVLGNVT   60
HXB2          TEKLWVTVYYGVPVWKEATTTLFCASDAKAYDTEVHNVWATHACVPTDPNPQEVVLVNVT   90
                 *************:* ************:** :*****************: * ***

YU2*          ENFNMWKNNMVEQMHEDIISLWDQSLKPCVKLTPLCVTLNCTDLRNATNTTSSS-----W   115
YU2dsm        ENFNMWKNNMVEQMHEDIISLRDQSLKPCVKLTPLCVTLNCTDLRNATNTTSSS-----W   115
JRFL*         EHFNMWKNNMVEQMQEDIISLWDQSLKPCVKLTPLCVTLNCKDVN-ATNTTNDS-----E   111
JRFLdsm       EHFNMWKNNMVEQMQEDIISLRDQSLKPCVKLTPLCVTLNCKDVN-ATNTTNDS-----E   111
BG505*        EEFNMWKNNMVEQMHTDIISLWDQSLKPCVKLTPLCVTLQCTNVTNNI------------   108
BG505SOSIP    EEFNMWKNNMVEQMHTDIISLWDQSLKPCVKLTPLCVTLQCTNVTNNI------------   108
BG505dsm      EEFNMWKNNMVEQMHTDIISLRDQSLKPCVKLTPLCVTLQCTNVTNNI------------   108
QH0692*       ENFNMWKNNMVEQMHEDIISLWDESLKPCVKLTPLCVTLNCTDEVKTSYANKTSNETYKT   120
QH0692dSOSIP  ENFNMWKNNMVEQMHEDIISLWDESLKPCVKLTPLCVTLNCTDEVKTSYANKTSNETYKT   120
QH0692dsm     ENFNMWKNNMVEQMHEDIISLRDESLKPCVKLTPLCVTLNCTDEVKTSYANKTSNETYKT   120
HXB2          ENFNMWKNDMVEQMHEDIISLWDQSLKPCVKLTPLCVSLKCTDLKNDTNTNSSSG----R   146
              *.******:*****: ***** *:*************:*:*.:

YU2*          ETMEKGEIKNCSFNITTSIRDKVQKEYALFYNLDVVPIDN------------ASYRLISC   163
YU2dsm        ETMEKGEIRICSFNITTSIRDKVRKEYALFYNLNVVPIDN------------ASHRLISC   163
JRFL*         GTMERGEIKNCSFNITTSIRDEVQKEYALFYKLDVVPIDNNN----------TSYRLISC   161
JRFLdsm       GTMERGEIRICSFNITTSIRDEVRKEYALFYKLNVVPIDNNN----------TSHRLISC   161
BG505*        TDDMRGELKNCSFNMTTELRDKKQKVYSLFYRLDVVQINENQGNRS--NNSNKEYRLINC   166
BG505SOSIP    TDDMRGELKNCSFNMTTELRDKKQKVYSLFYRLDVVQINENQGNRS--NNSNKEYRLINC   166
BG505dsm      TDDMRGELRICSFNMTTELRDKKRKVYSLFYRLNVVQINENQGNRS--NNSNKEHRLINC   166
QH0692*       SNETFGEIKNCSFSVPTGIKDKVQNVYALFYKLDVIPIDDNNNSSKNNNGSYSSYRLINC   180
QH0692dSOSIP  SNETFGEIKNCSFSVPTGIKDKVQNVYALFYKLDVIPIDDNNNSSKNNNGSYSSYRLINC   180
QH0692dsm     SNETFGEIRICSFSVPTGIKDKVRNVYALFYKLNVIPIDDNNNSSKNNNGSYSSHRLINC   180
HXB2          MIMEKGEIKNCSFNISTSIRGKVQKEYAFFYKLDIIPIDNDT----------TSYKLTSC   196
                   **:: ***.: * ::.: :: *::**.*::: *::             .::* .*

YU2*          NTSVITQACPKVSFEPIPIHYCAPAGFAILKCNDKKFNGTGPCTNVSTVQCTHGIRPVVS   223
YU2dsm        NTSVITQACPKVSFEPIPIHYCAPAGFAILKCNDKKFNGTGPCTNVSTVQCTHGIRPVVS   223
JRFL*         DTSVITQACPKISFEPIPIHYCAPAGFAILKCNDKTFNGKGPCKNVSTVQCTHGIRPVVS   221
JRFLdsm       DTSVITQACPKISFEPIPIHYCAPAGFAILKCNDKTFNGKGPCKNVSTVQCTHGIRPVVS   221
BG505*        NTSAITQACPKVSFEPIPIHYCAPAGFAILKCKDKKFNGTGPCPSVSTVQCTHGIKPVVS   226
BG505SOSIP    NTSAITQACPKVSFEPIPIHYCAPAGFAILKCKDKKFNGTGPCPSVSTVQCTHGIKPVVS   226
BG505dsm      NTSAITQACPKVSFEPIPIHYCAPAGFAILKCKDKKFNGTGPCPSVSTVQCTHGIKPVVS   226
QH0692*       NTSVITQACPKVSFEPIPIHYCAPAGFAILKCNNKTFNGTGPCTNVSTVQCTHGIRPVVS   240
QH0692dSOSIP  NTSVITQACPKVSFEPIPIHYCAPAGFAILKCNNKTFNGTGPCTNVSTVQCTHGIRPVVS   240
QH0692dsm     NTSVITQACPKVSFEPIPIHYCAPAGFAILKCNNKTFNGTGPCTNVSTVQCTHGIRPVVS   240
HXB2          NTSVITQACPKVSFEPIPIHYCAPAGFAILKCNNKTFNGTGPCTNVSTVQCTHGIRPVVS   256
              :**.*******:********************::*.***.*** .**********:****
```

Mathew *et al*., S1 Fig. (cont’d)

```
YU2*            TQLLLNGSLAEEEIVIRSENFTNNAKTIIVQLNESVVINCTRPNNNTRKSIN--IGPGRA      281
YU2dsm          TQLLLNGSLAEEEIVIRSENFTNNAKTIIVQLNESVVINCTRPNNNTRKSIN--IGPGRA      281
JRFL*           TQLLLNGSLAEEEVVIRSDNFTNNAKTIIVQLKESVEINCTRPNNNTRKSIH--IGPGRA      279
JRFLdsm         TQLLLNGSLAEEEVVIRSDNFTNNAKTIIVQLKESVEINCTRPNNNTRKSIH--IGPGRA      279
BG505*          TQLLLNGSLAEEEVMIRSENITNNAKNILVQFNTPVQINCTRPNNNTRKSIR--IGPGQA      284
BG505SOSIP      TQLLLNGSLAEEEVMIRSENITNNAKNILVQFNTPVQINCTRPNNNTRKSIR--IGPGQA      284
BG505dsm        TQLLLNGSLAEEEVMIRSENITNNAKNILVQFNTPVQINCTRPNNNTRKSIR--IGPGQA      284
QH0692*         TQLLLNGSLAEEEVVIRSENFTNNAKTIIVHLKKSVEINCTRPGNNTRKSIH--IGPGRA      298
QH0692dSOSIP    TQLLLNGSLAEEEVVIRSENFTNNAKTIIVHLKKSVEINCTRPGNNTRKSIH--IGPGRA      298
QH0692dsm       TQLLLNGSLAEEEVVIRSENFTNNAKTIIVHLKKSVEINCTRPGNNTRKSIH--IGPGRA      298
HXB2            TQLLLNGSLAEEEVVIRSVNFTDNAKTIIVQLNTSVEINCTRPNNNTRKRIRIQRGPGRA      316
                *************::*** *:*:***.*:*:::  * ******.***** *.   ***:*

YU2*            LYTTGEIIGDIRQAHCNLSKTQWENTLEQIAIKLKEQFGNNKTIIFNPSSGGDPEIVTHS      341
YU2dsm          LYTTGEIIGDIRQAHCNLSKTQWENTLEQIAIKLKEQYGNNKTIIFNPSSGGDPEIVTHS      341
JRFL*           FYTTGEIIGDIRQAHCNISRAKWNDTLKQIVIKLREQFEN-KTIVFNHSSGGDPEIVMHS      338
JRFLdsm         FYTTGEIIGDIRQAHCNISRAKWNDTLKQIVIKLREQYEN-KTIVFNHSSGGDPEIVMHS      338
BG505*          FYATGDIIGDIRQAHCTVSKATWNETLGKVVKQLRKHFGNNTIIRFANSSGGDLEVTTHS      344
BG505SOSIP      FYATGDIIGDIRQAHCNVSKATWNETLGKVVKQLRKHFGNNTIIRFANSSGGDLEVTTHS      344
BG505dsm        FYATGDIIGDIRQAHCNVSKATWNETLGKVVKQLRKHYGNNTIIRFANSSGGDLEVTTHS      344
QH0692*         FYATGDIIGDIRQAHCNLSSVQWNDTLKQIVIKLGEQFGTNKTIAFNQSSGGDPEIVMHS      358
QH0692dSOSIP    FYATGDIIGDIRQAHCNLSSVQWNDTLKQIVIKLGEQFGTNKTIAFNQSSGGDPEIVMHS      358
QH0692dsm       FYATGDIIGDIRQAHCNLSSVQWNDTLKQIVIKLGEQYGTNKTIAFNQSSGGDPEIVMHS      358
HXB2            FVTIGK-IGNMRQAHCNISRAKWNNTLKQIASKLREQFGNNKTIIFKQSSGGDPEIVTHS      375
                : : *. **::*****.:* . *::** ::. :* ::: . . * *  ***** *:. **

YU2*            FNCGGEFFYCNSTQLFT--WNDT-----RKLN-NTGRNITLPCRIKQIINMWQEVGKAMY      393
YU2dsm          FNCGGEFFYCNSTQLFT--WNDT-----RKLN-NTGRNITLPCRIKQINNMWQEVGKAMY      393
JRFL*           FNCGGEFFYCNSTQLFNSTWNNNTE---GSNN-TEGNTITLPCRIKQIINMWQEVGKAMY      394
JRFLdsm         FNCGGEFFYCNSTQLFNSTWNNNTE---GSNN-TEGNTITLPCRIKQINNMWQEVGKAMY      394
BG505*          FNCGGEFFYCNTSGLFNSTWISNTSV-QGSNSTGSNDSITLPCRIKQIINMWQRIGQAMY      403
BG505SOSIP      FNCGGEFFYCNTSGLFNSTWISNTSV-QGSNSTGSNDSITLPCRIKQIINMWQRIGQAMY      403
BG505dsm        FNCGGEFFYCNTSGLFNSTWISNTSV-QGSNSTGSNDSITLPCRIKQINNMWQRIGQAMY      403
QH0692*         FNCGGEFFYCNTTQLFNSTWEFHGNWTRSNFTESNSTTITLPCRIKQIVNMWQEVGKAMY      418
QH0692dSOSIP    FNCGGEFFYCNTTQLFNSTWEFHGNWTRSNFTESNSTTITLPCRIKQIVNMWQEVGKAMY      418
QH0692dsm       FNCGGEFFYCNTTQLFNSTWEFHGNWTRSNFTESNSTTITLPCRIKQINNMWQEVGKAMY      418
HXB2            FNCGGEFFYCNSTQLFNSTWFNSTWSTEGSNNTEGSDTITLPCRIKQIINMWQKVGKAMY      435
                ***********:: **.  *         . .   . .********** ****.:*:***

YU2*            APPIRGQIRCSSNITGLLLTRDGGKDTNGTEIFRPGGGDMRDNWRSELYKYKVVKIEPLG      453
YU2dsm          APPIRGQIRCSSNITGLLLTRDGGKDTNGTEIFRPGGGDMRDNWRSELYKYKVVKIEPLG      453
JRFL*           APPIRGQIRCSSNITGLLLTRDGGINENGTEIFRPGGGDMRDNWRSELYKYKVVKIEPLG      454
JRFLdsm         APPIRGQIRCSSNITGLLLTRDGGINENGTEIFRPGGGDMRDNWRSELYKYKVVKIEPLG      454
BG505*          APPIQGVIRCVSNITGLILTRDGGSTNSTTETFRPGGGDMRDNWRSELYKYKVVKIEPLG      463
BG505SOSIP      APPIQGVIRCVSNITGLILTRDGGSTNSTTETFRPGGGDMRDNWRSELYKYKVVKIEPLG      463
BG505dsm        APPIQGVIRCVSNITGLILTRDGGSTNSTTETFRPGGGDMRDNWRSELYKYKVVKIEPLG      463
QH0692*         APPIRGQIRCSSNITGLLLTRDGGVNG-TRETFRPGGGDMRDNWRSELYKYKVVKIEPLG      477
QH0692dSOSIP    APPIRGQIRCSSNITGLLLTRDGGVNG-TRETFRPGGGDMRDNWRSELYKYKVVKIEPLG      477
QH0692dsm       APPIRGQIRCSSNITGLLLTRDGGVNG-TRETFRPGGGDMRDNWRSELYKYKVVKIEPLG      477
HXB2            APPISGQIRCSSNITGLLLTRDGGNSNNESEIFRPGGGDMRDNWRSELYKYKVVKIEPLG      495
                **** * *** ******:******      * ****************************
```

Mathew *et al.*, S1 Fig. (cont'd)

```
YU2*          VAPTKAKRRVVQREK--RAVGLGALFLGFLGAAGSTMGAASITLTVQARQLLSGIVQQQN   511
YU2dsm        VAPTKCQRRVVQKREAEAATSTGATFSGFSGSAGSTMGATSITLTVQARQLLSGIVQQQN   513
JRFL*         VAPTKAQRRVVQKREAEA-VGIGAVFLGFLGAAGSTMGAASMTLTVQARLLLSGIVQQQN   513
JRFLdsm       VAPTKCQRRVVQKREAEAATSTGATFSGFSGSAGSTMGATSMTLTVQARLLLSGIVQQQN   514
BG505*        VAPTRAKRRVVGRRRRRRAVGIGAVFLGFLGAAGSTMGAASMTLTVQARNLLSGIVQQQS   523
BG505SOSIP    VAPTRCKRRVVGRRRRRRAVGIGAVFLGFLGAAGSTMGAASMTLTVQARNLLSGIVQQQS   523
BG505dsm      VAPTRCQRRVVGKREAEAATSTGATFSGFSGSAGSTMGATSMTLTVQARNLLSGIVQQQS   523
QH0692*       VAPTKAQRRVVQKREAEAATSTGATFSGFSGSAGSTMGATSITLTVQARQLLSGIVQQQN   537
QH0692dSOSIP  VAPTKCQRRVVQKREAEAATSTGATFSGFSGSAGSTMGATSITLTVQARQLLSGIVQQQN   537
QH0692dsm     VAPTKCQRRVVQKREAEAATSTGATFSGFSGSAGSTMGATSITLTVQARQLLSGIVQQQN   537
HXB2          VAPTKAKRRVVQREK--RAVGIGALFLGFLGAAGSTMGAASMTLTVQARQLLSGIVQQQN   553
              ****:.:**** :..    .. ** * ** *:*******:*:******* *********.

YU2*          NLLRAIEAQQHLLQLTVWGIKQLQARVLAVERYLRDQQLLGIWGCSGKLICTTTVPWNTS   571
YU2dsm        NLLRAPEAQQHLLQLTVWGIKQLQARVLAVERYLRDQQLLGIWGCSGKLICCTTVPWNTS   573
JRFL*         NLLRAIEAQQRMLQLTVWGIKQLQARVLAVERYLGDQQLLGIWGCSGKLICTTAVPWNAS   573
JRFLdsm       NLLRAPEAQQRMLQLTVWGIKQLQARVLAVERYLGDQQLLGIWGCSGKLICCTAVPWNAS   574
BG505*        NLLRAIEAQQHLLKLTVWGIKQLQARVLAVERYLRDQQLLGIWGCSGKLICTTNVPWNSS   583
BG505SOSIP    NLLRAPEAQQHLLKLTVWGIKQLQARVLAVERYLRDQQLLGIWGCSGKLICCTNVPWNSS   583
BG505dsm      NLLRAPEAQQHLLKLTVWGIKQLQARVLAVERYLRDQQLLGIWGCSGKLICCTNVPWNSS   583
QH0692*       NLLRAIEAQQHMLQLTVWGIKQLQARVLAVERYLRDQQLLGIWGCSGKLICTTAVPWNAS   597
QH0692dSOSIP  NLLRAPEAQQHMLQLTVWGIKQLQARVLAVERYLRDQQLLGIWGCSGKLICCTAVPWNAS   597
QH0692dsm     NLLRAPEAQQHMLQLTVWGIKQLQARVLAVERYLRDQQLLGIWGCSGKLICCTAVPWNAS   597
HXB2          NLLRAIEAQQHLLQLTVWGIKQLQARILAVERYLKDQQLLGIWGCSGKLICTTAVPWNAS   613
              ***** ****::*:************:******* **************** * ****:*

YU2*          WSNKSLNEIWDNMTWMKWEREIDNYTHIIYSLIEQSQNQQEKNEQELLALDKWASLWNWF   631
YU2dsm        WSNKSLNEIWDNMTWMKWEREIDNYTHIIYSLIEQSQNQQEKNEQELLALDKWASLWNWF   633
JRFL*         WSNKSLDRIWNNMTWMEWEREIDNYTSEIYTLIEESQNQQEKNEQELLELDKWASLWNWF   633
JRFLdsm       WSNKSLDRIWNNMTWMEWEREIDNYTSEIYTLIEESQNQQEKNEQELLELDKWASLWNWF   634
BG505*        WSNRNLSEIWDNMTWLQWDKEISNYTQIIYGLLEESQNQQEKNEQDLLALDKWASLWNWF   643
BG505SOSIP    WSNRNLSEIWDNMTWLQWDKEISNYTQIIYGLLEESQNQQEKNEQDLLALDKWASLWNWF   643
BG505dsm      WSNRNLSEIWDNMTWLQWDKEISNYTQIIYGLLEESQNQQEKNEQDLLALDKWASLWNWF   643
QH0692*       WSNKSQDYIWNNMTWMQWDKEINNYTNLIYSLLEDSQNQQEKNEHELLELDKWASLWNWF   657
QH0692dSOSIP  WSNKSQDYIWNNMTWMQWDKEINNYTNLIYSLLEDSQNQQEKNEHELLELDKWASLWNWF   657
QH0692dsm     WSNKSQDYIWNNMTWMQWDKEINNYTNLIYSLLEDSQNQQEKNEHELLELDKWASLWNWF   657
HXB2          WSNKSLEQIWNHTTWMEWDREINNYTSLIHSLIEESQNQQEKNEQELLELDKWASLWNWF   673
              ***:. . **:: **::*::**.***  *: *:*:*********::** ***********

YU2*          DITKWLWYIK   641
YU2dsm        DITKWLWYIK   643
JRFL*         DITKWLWY     641
JRFLdsm       DITKWLWY     642
BG505*        DISNWLWYIK   653
BG505SOSIP    DISNWLWYIK   653
BG505dsm      DISNWLWYIK   653
QH0692*       DITRWLWYI    666
QH0692dSOSIP  DITRWLWYI    666
QH0692dsm     DITRWLWYI    666
HXB2          NITNWLWYIK   683
              :*:.****
```
