# Supplementary figures and images for: Display of the HIV envelope protein at the yeast cell surface for immunogen development
Source: PLoS One. 2018 Oct 18;13(10):e0205756. doi: 10.1371/journal.pone.0205756 (PMC6193675; doi:10.1371/journal.pone.0205756)

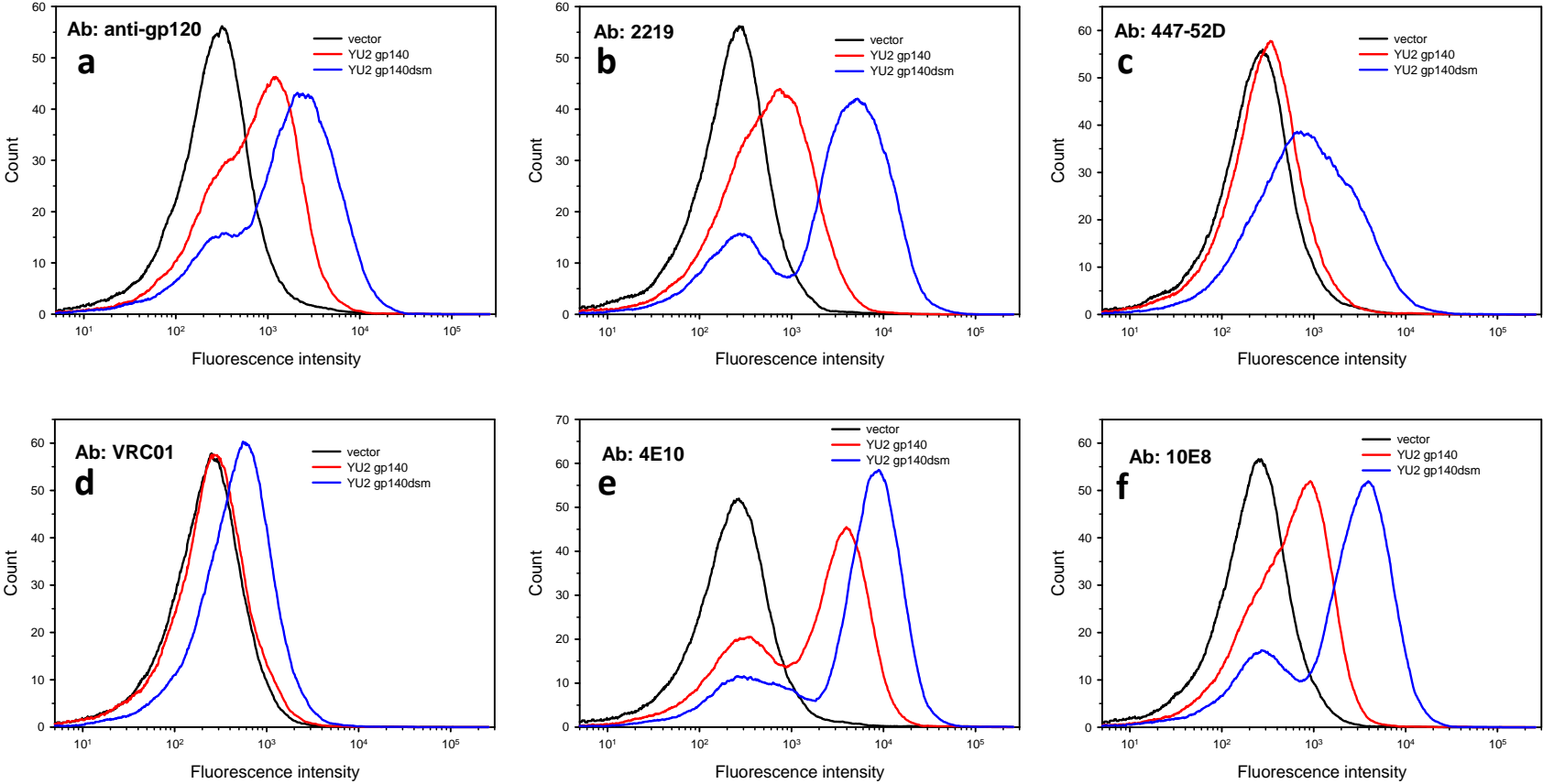

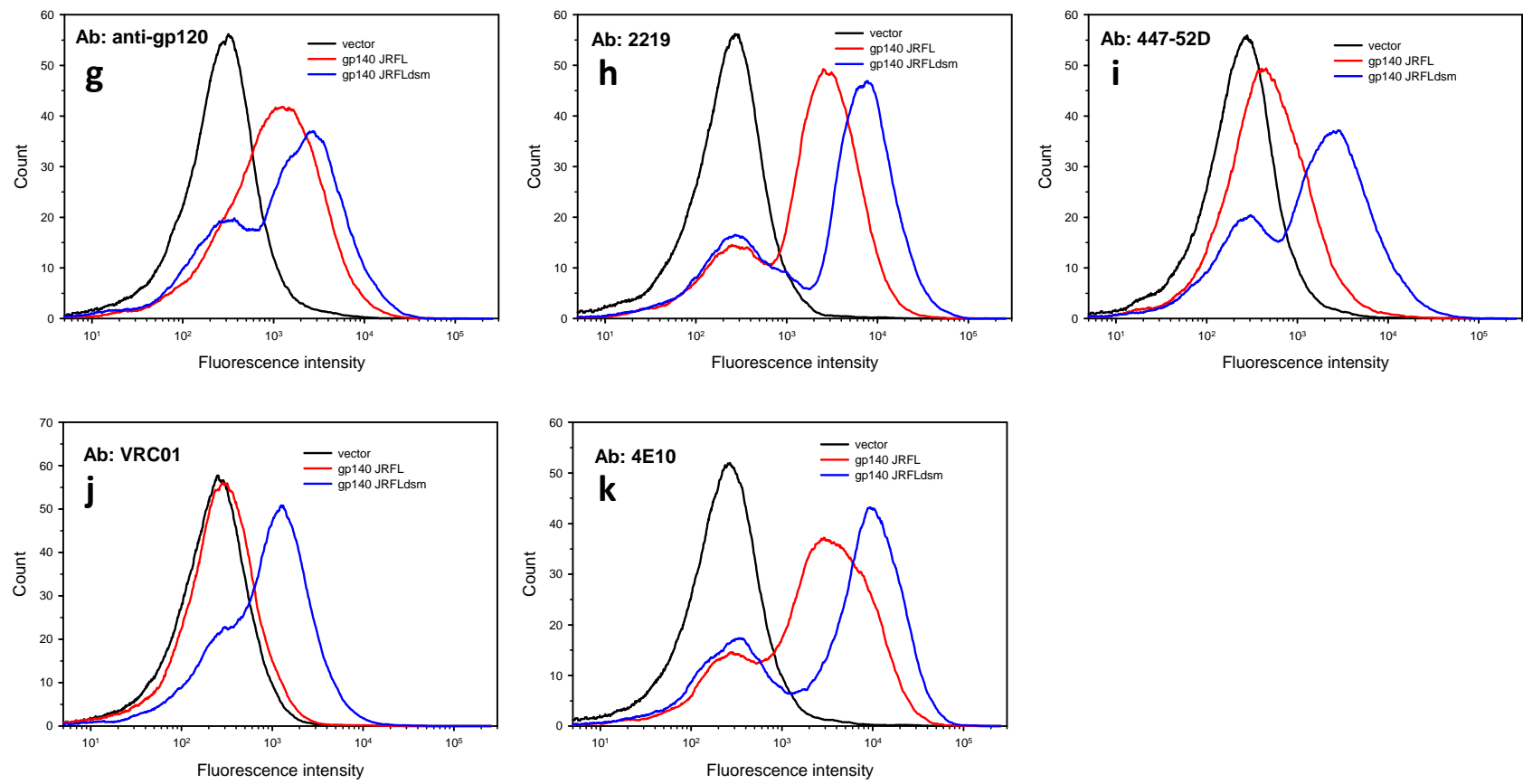

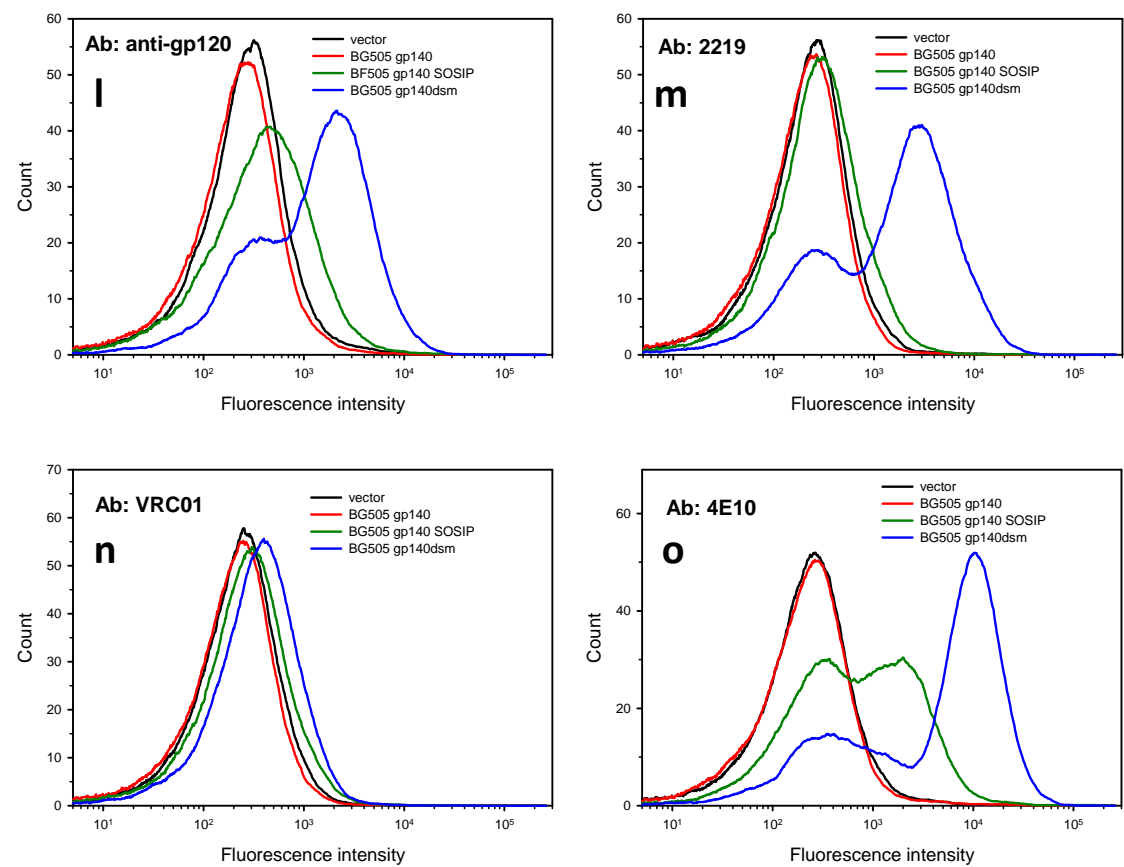

Supplement: S2 Fig — The figure shows a flow cytometry histogram of fluorescence of ~10,000 cells following incubation with the indicated primary antibodies and fluorescent secondary antibody. Panels (a)-(f) compared the unmodified and dsm forms of YU2 gp140. Panels (g)-(k) compared the unmodified and dsm forms of JRFL gp140. Panels (l)-(o) compared the unmodified, SOSIP (but without the dSOSIP mutations), and dsm forms of BG505 gp140. (a), (g), (l) were probed with anti-gp120 polyclonal antibody (~66 nM); (b), (h), and (m)) were probed with anti-V3 loop antibody 2219 (320 nM); (c) and (i) were probed with anti-V3 loop antibody 447-52D (90 nM); (d), (j), and (n) were probed with anti-CD4 binding site antibody VRC01 (340 nM); (e), (k), and (o) were probed with anti-MPER region antibody 4E10 (200 nM); (f) was probed with anti-MPER region antibody 10E8 (70 nM). (Secondary antibody incubations and flow cytometry were performed at room temperature). (PDF) [file pone.0205756.s002.pdf]

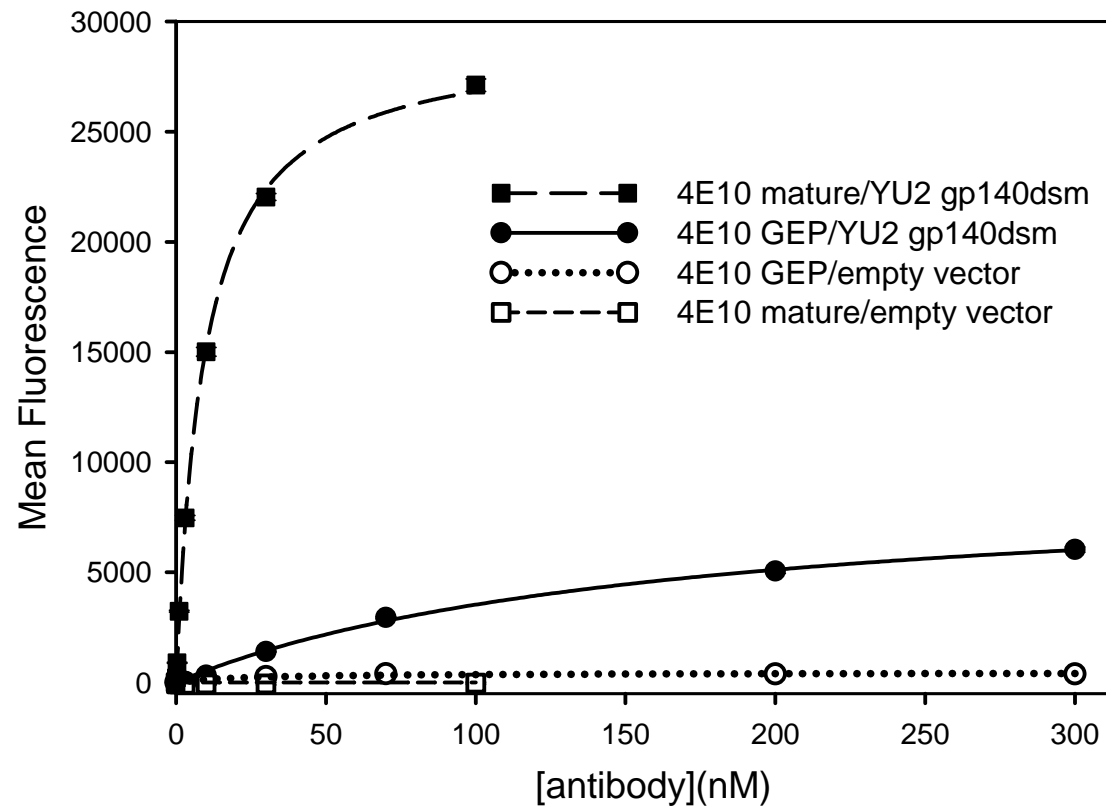

Supplement: S3 Fig — The indicated concentrations of antibodies were incubated with cells expressing different forms of Env as described in Materials and Methods. The binding assays were performed over different ranges of antibody concentrations for mature vs. precursor forms of 4E10 to facilitate fitting of binding curves in view of large differences in affinity. Error bars are smaller than the symbols. Based on triplicate biological replicates, the Kd for 4E10 mature binding to YU2 gp140dsm is 9.3 ± 0.4 and the relative Bmax is 29,300 ± 300. For 4E10 precursor binding to YU2 gp140dsm the Kd is 163 ± 8 nM and the relative Bmax is 9,300 ± 300. (PDF) [file pone.0205756.s003.pdf]
