# Supplementary material for: Display of the HIV envelope protein at the yeast cell surface for immunogen development
Source: PLoS One. 2018 Oct 18;13(10):e0205756. doi: 10.1371/journal.pone.0205756 (PMC6193675; doi:10.1371/journal.pone.0205756)
Supplement: S4 Fig — (PDF) [file pone.0205756.s004.pdf]

447-42D GL-Heavy chain

```
GAG GTG CAG CTG GTG GAG TCT GGG GGA GGC TTG GTA AAG CCT GGG GGG TCC CTT AGA CTC TCC TGT GCA GCC TCT GGT TTC ACT TTC AGT  < 90
E   V   Q   L   V   E   S   G   G   G   L   V   K   P   G   G   S   L   R   L   S   C   A   A   S   G   F   T   F   S

AAC GCC TGG ATG AAC TGG GTC CGC CAG GCT CCA GGG AAG GGG CTG GAG TGG GTC GGC CGT ATT AAA AGC AAA ACT GAT GGT GGG ACA ACA  < 180
N   A   W   M   N   W   V   R   Q   A   P   G   K   G   L   E   W   V   G   R   I   K   S   K   T   D   G   G   T   T

GAC TAC GCT GCA CCC GTG AAA GGC AGA TTC ACC ATC TCA AGA GAT GAT TCA AAA AAC ACG CTG TAT CTG CAA ATG AAC AGC CTG AAA ACC  < 270
D   Y   A   A   P   V   K   G   R   F   T   I   S   R   D   D   S   K   N   T   L   Y   L   Q   M   N   S   L   K   T

GAG GAC ACA GCC GTG TAT TAC TGT ACC ACA GAC GGT ATT ACT ATG GTT CGG GGA GTT AGT GAG GAT TAC TAC TAC TAC TAC ATG GAC GTC  < 360
E   D   T   A   V   Y   Y   C   T   T   D   G   I   T   M   V   R   G   V   S   E   D   Y   Y   Y   Y   Y   M   D   V

TGG GGC AAA GGG ACC ACG GTC ACC GTC TCC TCA  < 393
W   G   K   G   T   T   V   T   V   S   S
```

447-42D GL-Light chain

```
CAG TCT GTG TTG ACG CAG CCG CCC TCA GTG TCT GCG GCC CCA GGA CAG AAG GTC ACC ATC TCC TGC TCT GGA AGC AGC TCC AAC ATT GGG  < 90
Q   S   V   L   T   Q   P   P   S   V   S   A   A   P   G   Q   K   V   T   I   S   C   S   G   S   S   S   N   I   G

AAT AAT TAT GTA TCC TGG TAC CAG CAG CTC CCA GGA ACA GCC CCC AAA CTC CTC ATT TAT GAC AAT AAT AAG CGA CCC TCA GGG ATT CCT  < 180
N   N   Y   V   S   W   Y   Q   Q   L   P   G   T   A   P   K   L   L   I   Y   D   N   N   K   R   P   S   G   I   P

GAC CGA TTC TCT GGC TCC AAG TCT GGC ACG TCA GCC ACC CTG GGC ATC ACC GGA CTC CAG ACT GGG GAC GAG GCC GAT TAT TAC TGC GGA  < 270
D   R   F   S   G   S   K   S   G   T   S   A   T   L   G   I   T   G   L   Q   T   G   D   E   A   D   Y   Y   C   G

ACA TGG GAT AGC AGC CTG AGT GCT GGT TGG GTG TTC GGC GGA GGG ACC AAG CTG ACC GTC CTA  < 333
T   W   D   S   S   L   S   A   G   W   V   F   G   G   G   T   K   L   T   V   L
```

VRC01-GL-Heavy chain

```
ACC GGT GTA CAT TCC CAG GTG CAG CTG GTG CAG TCT GGG GCT GAG GTG AAG AAG CCT GGG GCC TCA GTG AAG GTC TCC TGC AAG GCT TCT  < 90
T   G   V   H   S   Q   V   Q   L   V   Q   S   G   A   E   V   K   K   P   G   A   S   V   K   V   S   C   K   A   S

GGA TAC ACC TTC ACC GGC TAC TAT ATG CAC TGG GTG CGA CAG GCC CCT GGA CAA GGG CTT GAG TGG ATG GGA TGG ATC AAC CCT AAC AGT  < 180
G   Y   T   F   T   G   Y   Y   M   H   W   V   R   Q   A   P   G   Q   G   L   E   W   M   G   W   I   N   P   N   S

GGT GGC ACA AAC TAT GCA CAG AAG TTT CAG GGC AGG GTC ACC ATG ACC AGG GAC ACG TCC ATC AGC ACA GCC TAC ATG GAG CTG AGC AGG  < 270
G   G   T   N   Y   A   Q   K   F   Q   G   R   V   T   M   T   R   D   T   S   I   S   T   A   Y   M   E   L   S   R

CTG AGA TCT GAC GAC ACG GCC GTG TAT TAC TGT GCG AGA GAC TAC GTG TGG GCT GAA TAC TTC CAG CAC TGG GGC CAG GGC ACC CTG GTC  < 360
L   R   S   D   D   T   A   V   Y   Y   C   A   R   D   Y   V   W   A   E   Y   F   Q   H   W   G   Q   G   T   L   V

ACC GTC TCC TCA GCG TCG ACC  < 381
T   V   S   S   A   S   T
```

VRC01-GL-Light chain

```
ACC GGT GTG CAC AGC GAA ATC GTC TTG ACG CAG TCG CCA GCG ACT CTT TCG CTT TCG CCG GGA GAA AGG GCG ACA CTT TCC TGT CGC GCG  < 90
T   G   V   H   S   E   I   V   L   T   Q   S   P   A   T   L   S   L   S   P   G   E   R   A   T   L   S   C   R   A

TCC CAG AGC GTA AGC AGC TAT CTG GCC TGG TAT CAG CAA AAA CCG GGA CAG GCA CCT CGC CTC CTG ATC TAC GAC GCC TCA AAC CGG GCG  < 180
S   Q   S   V   S   S   Y   L   A   W   Y   Q   Q   K   P   G   Q   A   P   R   L   L   I   Y   D   A   S   N   R   A

ACA GGG ATC CCG GCT AGA TTC TCC GGG TCA GGC TCG GGA ACA GAC TTC ACC CTC ACT ATT TCG TCA TTG GAG CCC GAG GAT TTT GCA GTC  < 270
T   G   I   P   A   R   F   S   G   S   G   S   G   T   D   F   T   L   T   I   S   S   L   E   P   E   D   F   A   V

TAT TAC TGC CAG CAG CGA TCA AAT TGG CCC TAC ACT TTT GGT CAA GGG ACG AAG CTG GAG ATT AAG CGT ACG  < 342
Y   Y   C   Q   Q   R   S   N   W   P   Y   T   F   G   Q   G   T   K   L   E   I   K   R   T
```

4E10-GL-Heavy chain

```
ACC GGT GTA CAT TCT CAG GTG CAG CTG GTG CAG TCT GGG GCT GAG GTG AAG AAG CCT GGG TCC TCG GTG AAG GTC TCC TGC AAG GCT TCT  < 90
T   G   V   H   S   Q   V   Q   L   V   Q   S   G   A   E   V   K   K   P   G   S   S   V   K   V   S   C   K   A   S

GGA GGC ACC TTC AGC AGC TAT GCT ATC AGC TGG GTG CGA CAG GCC CCT GGA CAA GGG CTT GAG TGG ATG GGA GGG ATC ATC CCT ATC TTT  < 180
G   G   T   F   S   S   Y   A   I   S   W   V   R   Q   A   P   G   Q   G   L   E   W   M   G   G   I   I   P   I   F

GGT ACA GCA AAC TAC GCA CAG AAG TTC CAG GGC AGA GTC ACG ATT ACC GCG GAC AAA TCC ACG AGC ACA GCC TAC ATG GAG CTG AGC AGC  < 270
G   T   A   N   Y   A   Q   K   F   Q   G   R   V   T   I   T   A   D   K   S   T   S   T   A   Y   M   E   L   S   S

CTG AGA TCT GAG GAC ACG GCC GTG TAT TAC TGT GCG AGA GAA GGG ACT ACT GGC TGG GGC TGG CTG GGC AAA CCC ATA GGG GCG TTT GCC  < 360
L   R   S   E   D   T   A   V   Y   Y   C   A   R   E   G   T   T   G   W   G   W   L   G   K   P   I   G   A   F   A

CAC TGG GGT CAA GGC ACT CTG GTC ACC GTC TCT TCA GCG TCG ACC  < 405
H   W   G   Q   G   T   L   V   T   V   S   S   A   S   T
```

4E10-GL-Light chain

```
ACC GGT GTA CAT TCA GAA ATT GTG TTG ACG CAG TCT CCA GGC ACC CTG TCT TTG TCT CCA GGG GAA AGA GCC ACC CTC TCC TGC AGG GCC  < 90
T   G   V   H   S   E   I   V   L   T   Q   S   P   G   T   L   S   L   S   P   G   E   R   A   T   L   S   C   R   A

AGT CAG AGT GTT TCC AGC AGC TAC TTA GCC TGG TAC CAG CAG AAA CCT GGC CAG GCT CCC AGG CTC CTC ATC TAT GGT GCA TCC AGC AGG  < 180
S   Q   S   V   S   S   S   Y   L   A   W   Y   Q   Q   K   P   G   Q   A   P   R   L   L   I   Y   G   A   S   S   R

GCA ACT GGC ATC CCA GAC AGG TTC AGT GGC AGT GGG TCT GGG ACA GAC TTC ACT CTC ACC ATC AGC AGA CTG GAG CCT GAA GAT TTT GCA  < 270
A   T   G   I   P   D   R   F   S   G   S   G   S   G   T   D   F   T   L   T   I   S   R   L   E   P   E   D   F   A

GTG TAT TAC TGT CAG CAG TAT GGT AGT TCA CTC TCC ACC TTC GGT CAA GGG ACC AAG GTG GAA GTC AAA CGT ACG  < 345
V   Y   Y   C   Q   Q   Y   G   S   S   L   S   T   F   G   Q   G   T   K   V   E   V   K   R   T
```
