# Supplementary material for: Display of the HIV envelope protein at the yeast cell surface for immunogen development
Source: PLoS One. 2018 Oct 18;13(10):e0205756. doi: 10.1371/journal.pone.0205756 (PMC6193675; doi:10.1371/journal.pone.0205756)
Supplement: S1 Table — For antibodies tested with an n of 2, both values are indicated. If the n is greater than 2, average values with SEMs are indicated. If the mean fluorescence after subtraction of the mean fluorescence of secondary plus cells was negative, it was assigned a value of zero. (PDF) [file pone.0205756.s005.pdf]

S1 Table. Mean fluorescence values used to determine the average values for Fig 4.

		Glycan	CD4i		CD4bs	Inter-face	V2 Glycan		V3 Glycan			V3 Loop						MPER				Nega-tive	Expres-sion
		2G12	17b	17b + sCD4	VRC01	CAP248-2B	PG9	PGT 145	10-1074	PGT 121	PGT 126	447-52D	2219	39F	268-D	2191	F425 B4e8	2F5	Z13e1	4E10	10E8	1069-D6	anti-V5
gp120	YU2	1392<br>1700	657<br>871	915<br>1157	2101 ±<br>26	69<br>0	6 ± 6	8 ± 5	1254<br>1256	95<br>0	14366<br>7963	42566<br>49301	49866<br>41371	2544<br>2715	193<br>516	58600<br>56646	56900<br>53965	12<br>0	4<br>3	16<br>0	1<br>0	4 ± 3	34248 ±<br>1854
gp120	mJRFL	3812<br>4573	8<br>0	352<br>256	8986 ±<br>830	162<br>59	0 ± 0	1 ± 1	4790<br>6640	291<br>103	2066<br>2585	26456<br>23430	22656<br>19049	2383<br>1700	25002<br>22036	25602<br>23608	28302<br>25362	8<br>0	39<br>0	44<br>0	21<br>0	5 ± 4	25949 ±<br>1715
gp120	mBG505	11295<br>11666	14<br>0	6<br>0	4449 ±<br>351	296<br>45	621 ±<br>90	10 ± 10	8250<br>8753	377<br>85	17952<br>10107	7654<br>9582	49252<br>37273	1918<br>1555	23<br>0	39584<br>36687	48284<br>43046	8<br>0	64<br>0	19<br>0	24<br>0	15 ± 13	29382 ±<br>2010
gp120	mYU2	1104<br>1139	0<br>0	516<br>449	6991 ±<br>363	116<br>91	51 ± 29	4 ± 4	3839<br>4009	275<br>0	12363<br>5835	49863<br>42228	49963<br>38432	2569<br>1787	201<br>302	45892<br>45998	44392<br>42806	0<br>0	0<br>0	0<br>13	0<br>0	31 ± 24	31980 ±<br>1422
gp120	mQH0692	12102<br>10993	7<br>0	155<br>197	3898 ±<br>171	134<br>35	0 ± 0	0 ± 0	11218<br>10113	359<br>24	16362<br>9280	49862<br>44711	48962<br>35198	2573<br>1878	51307<br>49251	50807<br>52648	56207<br>53452	18<br>7	15<br>0	44<br>0	1<br>0	9 ± 9	32600 ±<br>1524
gp140	YU2	103<br>57	15<br>0	11<br>0	2 ± 2	89<br>0	0 ± 0	0 ± 0	0<br>8	25<br>0	683<br>764	2586<br>1819	5368<br>4812	365<br>349	0<br>0	2222<br>2751	1593<br>1965	3476<br>1962	6152<br>4575	5909<br>3149	6109<br>4736	9 ± 4	5030 ±<br>193
gp140	mJRFL	2558<br>3412	5<br>0	29<br>0	3828 ±<br>290	158<br>62	32 ± 28	2 ± 2	2618<br>3363	41<br>41	2675<br>3045	22460<br>18435	21560<br>16197	1260<br>946	22795<br>18195	23495<br>20019	28195<br>23453	20505<br>13666	28505<br>20688	20805<br>13197	6148<br>4831	5 ± 3	11657 ±<br>618
gp140	mBG505	3549<br>4127	5<br>0	0<br>21	1224 ±<br>190	106<br>0	173 ±<br>36	0 ± 0	2354<br>3152	78<br>0	4710<br>4809	505<br>535	18451<br>13375	457<br>840	14<br>3	9297<br>10739	14238<br>15025	11663<br>7783	17763<br>16256	17863<br>10115	2892<br>3275	3 ± 3	12554 ±<br>469
gp140	mYU2	490<br>435	3<br>58	57<br>20	1575 ±<br>38	113<br>0	4 ± 4	12 ± 12	824<br>886	22<br>0	4617<br>4447	21154<br>17733	25654<br>19438	1331<br>1334	7<br>0	19284<br>18741	14184<br>14060	14074<br>7529	22974<br>15708	17974<br>7727	23374<br>17157	2 ± 1	14290 ±<br>730
gp140	mQH0692	5447<br>5116	9<br>0	8<br>0	1440 ±<br>200	79<br>25	4 ± 4	9 ± 9	5345<br>6122	89<br>0	11256<br>6857	27756<br>31660	32856<br>25452	1759<br>1471	36078<br>34029	38178<br>37852	42778<br>41418	18382<br>14389	22282<br>15495	16482<br>9885	1038<br>974	28 ± 13	10752 ±<br>799
	Empty vector	87<br>73	5<br>28	2<br>14	2±2	103<br>0	3 ± 3	19 ± 14	0<br>33	28<br>5	89<br>0	31<br>0	14<br>0	6<br>0	0<br>0	0<br>2	0<br>0	0<br>0	0<br>25	0<br>8	18<br>21	4 ± 2	33593 ±<br>2330
	n	2	2	2	3	2	3	4	2	2	2	2	2	2	2	2	2	2	2	2	2	10	10
