# Supplementary material for: Display of the HIV envelope protein at the yeast cell surface for immunogen development
Source: PLoS One. 2018 Oct 18;13(10):e0205756. doi: 10.1371/journal.pone.0205756 (PMC6193675; doi:10.1371/journal.pone.0205756)
Supplement: S2 Table — For each tested antibody a one-way ANOVA was performed followed by Dunnett’s post-test of the p value for deciding whether mean fluorescence intensities for each of the indicated viral strains are significantly different from the fluorescence values measured for empty vector tested with the same antibody. Because of the extreme differences in variance between measurements at low vs. high fluorescence values, the analysis of significance was performed on fluorescence values transformed by logarithmic transformation to equalize variances, after addition of a value of 200 to each fluorescence value (to accommodate negative fluorescence values following background subtraction of samples lacking primary antibody). The analysis was performed using Graphpad Prism software. Cells with grey shading indicate p values less than 0.05. (PDF) [file pone.0205756.s006.pdf]

S2 Table. Calculated p values for binding data presented in Fig 4.

		Glycan	CD4i		CD4bs	Inter-face	V2 Glycan		V3 Glycan			V3 Loop						MPER				Negative
		2G12	17b	17b + sCD4	VRC01	CAP 248-2B	PG9	PGT 145	10-1074	PGT 121	PGT 126	447-52D	2219	39F	268-D	2191	F425 B4e8	2F5	Z13e1	4E10	10e8	1069-D6
gp120	YU2	P < 0.01	P < 0.01	P < 0.01	P < 0.01	P > 0.05	P > 0.05	P > 0.05	P < 0.01	P > 0.05	P < 0.01	P < 0.01	P < 0.01	P < 0.01	P < 0.01	P < 0.01	P < 0.01	P > 0.05	P > 0.05	P > 0.05	P > 0.05	P > 0.05
	mJRFL	P < 0.01	P > 0.05	P < 0.01	P < 0.01	P > 0.05	P > 0.05	P > 0.05	P < 0.01	P > 0.05	P < 0.01	P < 0.01	P < 0.01	P < 0.01	P < 0.01	P < 0.01	P < 0.01	P > 0.05	P > 0.05	P > 0.05	P > 0.05	P > 0.05
	mBG505	P < 0.01	P > 0.05	P > 0.05	P < 0.01	P > 0.05	P < 0.01	P > 0.05	P < 0.01	P > 0.05	P < 0.01	P < 0.01	P < 0.01	P < 0.01	P > 0.05	P < 0.01	P < 0.01	P > 0.05	P > 0.05	P > 0.05	P > 0.05	P > 0.05
	mYU2	P < 0.01	P > 0.05	P < 0.01	P < 0.01	P > 0.05	P > 0.05	P > 0.05	P < 0.01	P > 0.05	P < 0.01	P < 0.01	P < 0.01	P < 0.01	P < 0.01	P < 0.01	P < 0.01	P > 0.05	P > 0.05	P > 0.05	P > 0.05	P > 0.05
	mQH0692	P < 0.01	P > 0.05	P < 0.05	P < 0.01	P > 0.05	P > 0.05	P > 0.05	P < 0.01	P > 0.05	P < 0.01	P < 0.01	P < 0.01	P < 0.01	P < 0.01	P < 0.01	P < 0.01	P > 0.05	P > 0.05	P > 0.05	P > 0.05	P > 0.05
gp140	YU2	P > 0.05	P > 0.05	P > 0.05	P > 0.05	P > 0.05	P > 0.05	P > 0.05	P > 0.05	P > 0.05	P < 0.01	P < 0.01	P < 0.01	P < 0.01	P > 0.05	P < 0.01	P < 0.01	P < 0.01	P < 0.01	P < 0.01	P < 0.01	P > 0.05
	mJRFL	P < 0.01	P > 0.05	P > 0.05	P < 0.01	P > 0.05	P > 0.05	P > 0.05	P < 0.01	P > 0.05	P < 0.01	P < 0.01	P < 0.01	P < 0.01	P < 0.01	P < 0.01	P < 0.01	P < 0.01	P < 0.01	P < 0.01	P < 0.01	P > 0.05
	mBG505	P < 0.01	P > 0.05	P > 0.05	P < 0.01	P > 0.05	P < 0.01	P > 0.05	P < 0.01	P > 0.05	P < 0.01	P < 0.01	P < 0.01	P < 0.01	P > 0.05	P < 0.01	P < 0.01	P < 0.01	P < 0.01	P < 0.01	P < 0.01	P > 0.05
	mYU2	P < 0.01	P > 0.05	P > 0.05	P < 0.01	P > 0.05	P > 0.05	P > 0.05	P < 0.01	P > 0.05	P < 0.01	P < 0.01	P < 0.01	P < 0.01	P > 0.05	P < 0.01	P < 0.01	P < 0.01	P < 0.01	P < 0.01	P < 0.01	P > 0.05
	mQH0692	P < 0.01	P > 0.05	P > 0.05	P < 0.01	P > 0.05	P > 0.05	P > 0.05	P < 0.01	P > 0.05	P < 0.01	P < 0.01	P < 0.01	P < 0.01	P < 0.01	P < 0.01	P < 0.01	P < 0.01	P < 0.01	P < 0.01	P < 0.01	P > 0.05
