# Supplementary material for: Display of the HIV envelope protein at the yeast cell surface for immunogen development
Source: PLoS One. 2018 Oct 18;13(10):e0205756. doi: 10.1371/journal.pone.0205756 (PMC6193675; doi:10.1371/journal.pone.0205756)
Supplement: S3 Table — (PDF) [file pone.0205756.s007.pdf]

S3 Table. Oligonucleotides and procedures for construction of different Env-expressing plasmids.

SAG1				
**Yeast host Strain is BCY123**				
Strain	Plasmid	Primer	Primer Sequence	Description/Notes
-	-	ON1455	AAA AAG CTT CTT ACA ACA AAT ATA CCA AAA TGA GAT TTC CTT CAA TTT TTA CTG CTG TTT TAT TCG	Primer pair to PCR the α-factor secretion signal from plasmid pPICZαA (Invitogen). The primers incorporate 5' HindIII and 3' EcoRI sites.
		ON1456	GGG CCA CGT GAA TTC AGC TTC AGC C	
-	-	ON1457	AAA GAA TTC GCG GCC GCT GCA TGC TGC TAA AAG CTC TTT TAT CTC AAC CAC TAC TAC TGA TTT AAC AAG	Primer pair to PCR SAG1 C-terminal region from the yeast chromosome. The primers incorporate 5' EcoRI and 3' XbaI sites.
		ON1458	ATT TCT AGA TTA GAA TAG CAG GTA CGA CAA AAG CAG AAA AAT GAT C	
A4605	pMD1918	-	-	Vector containing *GAL1* promoter, pre-pro alpha signal sequence and gene for the cell wall anchoring region of Sag1p. Details of the construction are described in the Materials and Methods section.
A4693	pMD2359	ON1524	AAACTCGAGAAAAGAGGTAAGCCAATTCCAAATCCATTGTTGGGTTTAGATTCTACTGCTGAACAATTGTGGGTTACAGTATATTACG	Primer pair to amplify and subclone V5 tag-YU2 gp140 into plasmid pMD1918 digested with XhoI and SphI in order to make plasmid pMD2359
		ON1525	TTTGCATGCTTGATGTACCACAACCACTTTGTGATG	
A4766	pMD2426	ON1524	AAACTCGAGAAAAGAGGTAAGCCAATTCCAAATCCATTGTTGGGTTTAGATTCTACTGCTGAACAATTGTGGGTTACAGTATATTACG	Primer pair to amplify and subclone V5 tag-YU2 gp120 with a mutagenized furin cleavage site into plasmid pMD1918digested with XhoI and SphI in order to make plasmid pMD2426
		ON1584	TTTGCATGCTTCTTTTCGGATTGAACGACAGAGGACTTTGC	

AGA2				
**Yeast host strain is EBY100**				
A4793	pYD5	-	-	Empty vector- negative control for C-terminal Aga2p fusions [1]
A4794	pMD2388	ON1544	GATCGGCTAGCGTACTGGCGGCGGGGGCTGAACAATTGTGGGTTACAGTATATTACG	Primer pair to amplify and subclone YU2 gp140 into plasmid pYD5 digested with NheI and EcoRI in order to make plasmid pMD2388
		ON1546	TTGTGGAATTCCTTGATGTACCACAACCACTTTGTGATG	
A4799	pYD1	-	-	Empty vector- negative control for N-terminal Aga2p fusions [2]
A4800	pMD2395	ON1535	TTTAAGCTTGCTGAACAATTGTGGGTTACAGTATATTACG	Primer pair to amplify and subclone YU2 gp120/HindIII (partial digest)/BstBI into pYD1/HindIII/BstBI in order to make plasmid pMD2395
		ON1537	AATTTCGAATCTCTTTTCTCTTTGAACGACTCTTCTCTTTG	
A4801	pMD2411	ON1544	GATCGGCTAGCGTACTGGCGGCGGGGGCTGAACAATTGTGGGTTACAGTATATTACG	

		ON1587	TTGTGGAATTCAGACTTTTCAGATTGAACGACGG AAGACTTTGCTTTTGTAGGAGCAACACCTAATG	Primer pair to amplify and subclone YU2 gp120 with a mutagenized furin cleavage site into plasmid pYD5 in order to make plasmid pMD2411
A4943	pMD2569	ON1681	GATCGGCTAGCGTACTGGCGGCGGGGGCTGAA AACTTATGGGTCACTGTCTATTACG	Primer pair to amplify and subclone BG505 gp140 SOSIP with R6 as the furin cleavage site into plasmid pYD5 digested with NheI and EcoRI in order to make plasmid pMD2569
		ON1684	TTGTGGAATTCCTTGATGTACCACAACCAGTTAG AAATATCGAAC	
A5017	pMD2592	ON1681	GATCGGCTAGCGTACTGGCGGCGGGGGCTGAA AACTTATGGGTCACTGTCTATTACG	Primer pair to amplify and subclone BG505 gp140 with R6 as the furin cleavage site into plasmid pYD5 digested with NheI and EcoRI in order to make plasmid pMD2592
		ON1684	TTGTGGAATTCCTTGATGTACCACAACCAGTTAG AAATATCGAAC	
A5073	pMD2636	ON1744	GATCATATTCGCTAGCGCTCAAGTTTCTGCCGCT GAAG	Primer pair to amplify and subclone streptavidin into plasmid pYD5 digested with NheI and EcoRI in order to make plasmid pMD2636
		ON1745	TTTCATGATCGAATTCAGAAGCAGCAGATGGCTT GACC	
	pMD2645	ON1751	AAATTGTAAACGTTAATATTTTGTTAAAATTCGC GTTAAATTTTT	Primer pair to amplify F1 ori from plasmid pMD1918. It was then subcloned into the PmeI site of plasmid pYD5. There is no difference in expression of the Aga2p fusion genes in cells transformed with pYD5 versus pMD2645 (not shown). The F1 ori allows for ssDNA isolation for site-directed mutagenesis.
		ON1752	AAACACGCGCCCTGTAGC	
A5146	pMD2667	-	-	Directly subcloned the NheI and EcoRI-digested JRFL gp140dsm gBlock into plasmid pYD5 digested with NheI and EcoRI in order to make plasmid pMD2667
A5157	pMD2672	ON1784	TATATGCTAGCGTACTGGCGGCGGGGGCAGAAA ATCTTTGGGTCACTGTGTATTATGG	Primer pair to amplify and subclone QH0692 gp140 into plasmid pYD5 digested with NheI and EcoRI in order to make plasmid pMD2672
		ON1786	GCGGCGGAATTCTATATACCACAACCATCTTGTG ATGTCAAACCAATTCCACAAAGAAGC	
A5158	pMD2673	ON1784	TATATGCTAGCGTACTGGCGGCGGGGGCAGAAA ATCTTTGGGTCACTGTGTATTATGG	Primer pair to amplify and subclone QH0692 gp140 dSOSIP into plasmid pYD5 digested with NheI and EcoRI in order to make plasmid pMD2673
		ON1786	GCGGCGGAATTCTATATACCACAACCATCTTGTG ATGTCAAACCAATTCCACAAAGAAGC	
A5159	pMD2674	ON1784	TATATGCTAGCGTACTGGCGGCGGGGGCAGAAA ATCTTTGGGTCACTGTGTATTATGG	Primer pair to amplify and subclone QH0692 gp140dsm into plasmid pYD5 digested with NheI and EcoRI in order to make plasmid pMD2674
		ON1786	GCGGCGGAATTCTATATACCACAACCATCTTGTG ATGTCAAACCAATTCCACAAAGAAGC	
A5212	pMD2684	ON1790	GATATTCGGCTAGCGTACTGGCGGC	Primer pair to amplify and subclone JRFL gp120dsm with a mutagenized furin cleavage site into plasmid pYD5 digested with NheI and EcoRI in order to make plasmid pMD2684
		ON1820	CTCAACTCGAATTCAGACTTTTGCACAACAGAAG ACTGACATTTTGTTGGCGCCACAC	
A5213	pMD2685	ON1784	TATATGCTAGCGTACTGGCGGCGGGGGCAGAAA ATCTTTGGGTCACTGTGTATTATGG	Primer pair to amplify and subclone QH0692 gp120 with a mutagenized furin cleavage site into plasmid pYD5 digested with NheI and EcoRI in order to make plasmid pMD2685
		ON1821	CGGCGGAATTCAGACTTTTGGACGACAGAAGAT TGAGCCTTGGTAGGCGCCA	

A5214	pMD2686	ON1784	TATATGCTAGCGTACTGGCGGCGGGGGCAGAAA ATCTTTGGGTCACTGTGTATTATGG	Primer pair to amplify and subclone QH0692 gp120 dSOSIP with a mutagenized furin cleavage site into plasmid pYD5 digested with NheI and EcoRI in order to make plasmid pMD2686
		ON1822	CGGCGGAATTCAGACTTTTGGACGACAGAAGAT TGACACTTGGTAGGCGCCA	
A5215	pMD2687	ON1784	TATATGCTAGCGTACTGGCGGCGGGGGCAGAAA ATCTTTGGGTCACTGTGTATTATGG	Primer pair to amplify and subclone QH0692 gp120dsm with a mutagenized furin cleavage site into plasmid pYD5 digested with NheI and EcoRI in order to make plasmid pMD2687
		ON1822	CGGCGGAATTCAGACTTTTGGACGACAGAAGAT TGACACTTGGTAGGCGCCA	
A5216	pMD2689	-	-	QH0692 gp120dsm with a G459V mutation (found this spontaneously mutated version when confirming construction of plasmid pMD2687)
		-	-	
A5235	pMD2713	-	-	Directly subcloned the NheI and EcoRI-digested JRFL gp140 gBlock (with amino acids KREAE substituted for the furin cleavage site REKR) into plasmid pMD2645 digested with NheI and EcoRI in order to make plasmid pMD2713
A5236	pMD2714	ON1544	GATCGGCTAGCGTACTGGCGGCGGGGGCTGAAC AATTGTGGGTTACAGTATATTACG	Primer pair to amplify and subclone YU2 gp120dsm with a mutagenized furin cleavage site into plasmid pMD2645 digested with NheI and EcoRI in order to make plasmid pMD2714
		ON1587	TTGTGGAATTCAGACTTTTCAGATTGAACGACGG AAGACTTTGCTTTTGTAGGAGCAACACCTAATG	
A5237	pMD2715	ON1544	GATCGGCTAGCGTACTGGCGGCGGGGGCTGAAC AATTGTGGGTTACAGTATATTACG	Primer pair to amplify and subclone YU2 gp140dsm into plasmid pMD2645 digested with NheI and EcoRI in order to make plasmid pMD2715
		ON1546	TTGTGGAATTCCTTGATGTACCACAACCACTTTGT GATG	
A5238	pMD2716	ON1681	GATCGGCTAGCGTACTGGCGGCGGGGGCTGAA AACTTATGGGTCACTGTCTATTACG	Primer pair to amplify and subclone BG505 gp120dsm with a mutagenized furin cleavage site into plasmid pMD2645 digested with NheI and EcoRI in order to make plasmid pMD2716
		ON1683	TTGTGGAATTCACCAACGACGGAAGACTTACATC TAGTTGGGGCAACACC	
A5239	pMD2717	ON1681	GATCGGCTAGCGTACTGGCGGCGGGGGCTGAA AACTTATGGGTCACTGTCTATTACG	Primer pair to amplify and subclone BG505 gp140dsm into plasmid pMD2645 digested with NheI and EcoRI in order to make plasmid pMD2715
		ON1684	TTGTGGAATTCCTTGATGTACCACAACCAGTTAG AAATATCGAAC	
		ON1966	GCTTCTTTGTGGAATGCTGCCGACATCGCTAGAT GGTTGTGGTAT	Forward primer to mutagenize W672A F673A T676A of QH0692 dsm gp140

Supporting Table S6 References:

1. Wang Z, Mathias A, Stavrou S, Neville DM, Jr. A new yeast display vector permitting free scFv amino termini can augment ligand binding affinities. Protein Eng Des Sel. 2005;18(7):337-43. doi: 10.1093/protein/gzi036. PubMed PMID: 15976011.
2. Kieke MC, Cho BK, Boder ET, Kranz DM, Wittrup KD. Isolation of anti-T cell receptor scFv mutants by yeast surface display. Protein Eng. 1997;10(11):1303-10. PubMed PMID: 9514119.
